# Energy Emissions Accounting Methods Can Determine Whether Direct Air Capture with Storage Achieves Net Removal

**DOI:** 10.1021/acs.est.5c13494

**Published:** 2026-04-02

**Authors:** Rebecca J. Hanes, Keju An, Wilson McNeil, Yijin Li, Isaias Marroquin, Soomin Chun, Sarah L. Nordahl, Kimberley K. Mayfield, Sarah E. Baker, Corinne D. Scown, Evan D. Sherwin

**Affiliations:** † National Laboratory of the Rockies, Golden, Colorado 80401, United States; ‡ 1666 Lawrence Berkeley National Laboratory, Berkeley, California 94720, United States; § Life-Cycle, Economics, and Agronomy Division, Joint BioEnergy Institute, Lawrence Berkeley National Laboratory, Emeryville, California 94608, United States; ∥ Lawrence Livermore National Laboratory, Livermore, California 94550, United States; ⊥ Energy and Biosciences Institute, 1438 University of California, Berkeley, California 94720, United States

**Keywords:** carbon dioxide removal, direct air capture, carbon accounting, electricity emissions, marginal emissions

## Abstract

The voluntary carbon market within the United States has expanded rapidly in recent years and enabled private companies and other organizations to provide revenue streams to carbon dioxide removal (CDR) technologies. For a CDR technology to participate in the voluntary carbon market (VCM), the emissions associated with constructing and operating the technology must be less than the CO_2_ captured from the atmosphere. Assessing the extent to which this is true for direct air capture with storage (DACS), a relatively energy-intensive CDR technology, strongly depends on the accounting method used to assess the emissions intensity of purchased energy. We simulate the hourly weather-dependent operation of sorbent- and solvent-based DACS in California, Louisiana, Texas, and Wyoming, representing a wide range of local weather and electric and natural gas grid compositions. In all cases, the single most important emissions accounting decision is the method used to estimate the emissions intensity of purchased grid electricity, which varies the calculated net removal by −1049% to +108%. All other factors influencing net removal introduce a variation of at most ±14%. No electricity emissions accounting method is universally conservative across all scenarios, and none is objectively more accurate. High-spatiotemporal-resolution, high-quality, publicly available data sets and models for electricity emissions accounting do not currently exist and are urgently needed to enable standardization of emissions accounting methods to more accurately determine the true emissions impacts of DACS and other energy-intensive facilities.

## Graphical abstract

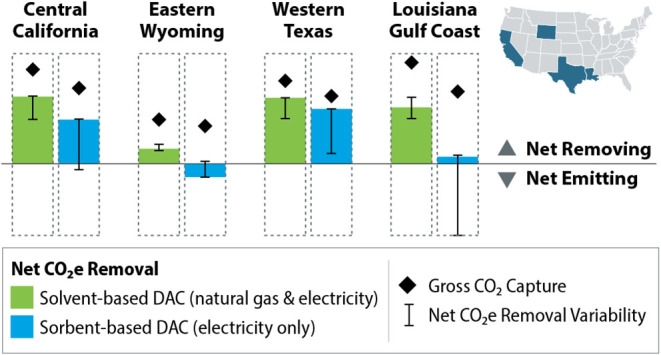

## Introduction

Direct air capture with storage (DACS)^1,2^ is a developing carbon dioxide removal (CDR) technology with potential to supply the United States’ and global voluntary carbon market^3–5^ (VCM). The VCM and related private sector purchasing mechanisms currently constitute the vast majority of global DACS deployment.^3^ To enable DACS developers to participate in the VCM, DACS systems must be able to show positive net CO_2_e removal, which is the difference between CO_2_ captured and durably stored by the system, and the CO_2_e emissions released as a result of the system operating. Net CO_2_e removal for energy-intensive DACS systems depends heavily on the emissions intensities of input energy carriers,^6–9^ including purchased grid electricity and natural gas.^10^ The same is true for other emissions-conscious, energy-intensive industries such as green hydrogen,^11^ data centers,^12,13^ and many heavy industrial processes.

Emissions accounting for purchased energy aims to quantify the emissions caused by a facility’s energy purchases. However, determining causality across the electric grid, a complex and dynamic network, is extremely challenging and requires developing a credible proxy for a value that fundamentally cannot be measured or observed: the proportion of electrons entering a facility that originated at specific grid generators. We compare five methods that approximate this quantity. The most common method for electricity emissions accounting uses annual volumetric matching, assuming that purchased electricity has the average emissions intensity of all electricity produced throughout the year in the region or country in question. This method is recommended in current standards for calculating DACS net removal,^14,15^ and is the method most commonly used in attributional life cycle assessment (LCA) studies. High-quality, peer-reviewed, regularly updated data is publicly available in the United States for annual average electricity emissions accounting calculations, albeit at a coarse spatial resolution and with a multiyear time delay.^16^ However, annual average accounting does not capture subannual variations in electricity emissions intensity, which can be substantial in regions with seasonal swings in renewable electricity generation. Fluctuating electricity demand, such as for DACS facilities^17^ and data centers,^18^ leads to further variability in electricity emissions. Electricity demands that are new to the grid or that fluctuate can also incur marginal impacts, which capture the grid response to the demand. Short-run marginal emission factors are calculated assuming that additional electricity demand is met by the lowest-cost dispatchable resource within the existing fleet of generators and reflect operational changes to the grid.^19–21^ Long-run marginal emission factors represent new generators added to the grid over time to meet sustained additional electricity demand.^22,23^ Marginal emission factors, unlike average factors, are generally calculated using grid models because primary data cannot indicate which generators would have been dispatched or added under a hypothetical scenario (the additional demand being assessed) that did not occur.

In this work, we calculate annual net CO_2_e removal achieved by sorbent- and solvent-based DACS facilities in the U.S. and assess the variation in net removal caused by applying five methods for calculating emissions associated with purchased grid electricity. This represents the first side-by-side comparison of different electricity emissions accounting methods applied to variable electricity demands at an hourly resolution. We employ the Regional Energy Deployment System (ReEDS) capacity expansion model^24,25^ and the Cambium hourly model^19,26^ to obtain hourly average, short-run, and long-run marginal emission factors, and to calculate annual average factors, for empirically modeled^17,27^ DACS facilities operating in a single year, 2022. As a comparison to standard accounting methods, we also apply a method that uses annual average emissions factors derived from primary grid mix data^16^ and generator-specific emissions factors.^28^ We also qualitatively discuss the emissions effects of two alternative powering scenarios: dedicated on-site renewable generation and rigorous power purchasing agreements (PPAs). A full assessment of the technological and economic viability of these powering alternatives is outside the scope of this analysis.

We assess net removal variability for DACS facilities in four locations, central California, the Louisiana Gulf Coast, western Texas, and eastern Wyoming, to capture the impact of the local grid on electricity emissions and the impact of local weather conditions on DACS efficiency. To highlight the relative importance of electricity emissions accounting methods alongside other factors in calculating net removal, we conduct a sensitivity analysis on the embodied emissions for nonelectricity purchased inputs, including natural gas, sorbent and solvent materials, and water, and on facility operational scenarios.

We find that the method used to calculate the emissions intensity of purchased grid electricity is the single most impactful factor in calculating the net removal of CO_2_e from a DACS facility. This finding holds across DACS technologies, local weather conditions, facility operational scenarios, and electric grid mixes. Our results highlight the urgent need for standardization of energy emissions accounting methods for CDR technologies, as well as the build-out of high-quality, high-spatiotemporal-resolution, publicly available data sets and models for electricity emissions accounting to help determine the true emissions impacts of DACS and other energy-intensive facilities.

## Materials and Methods

Calculating net CO_2_e removal for a DACS facility requires quantifying numerous direct and embodied emissions (Figure 1). For this analysis, we set an expansive system boundary with the intention of calculating the net CO_2_e removal attributable to the construction and operation of the DACS facility and infrastructure.^29^ We quantify gross CO_2_ capture by DACS facilities, emissions associated with the energy used at the facility, on-site emissions, and embodied emissions associated with the construction and operation of the facility. We exclude from this analysis fluxes of smaller magnitude such as land use change^1^ and direct CO_2_ leakage from the DACS facility^30^ and fluxes without established quantification methods such as economic leakage. Geological CO_2_ storage in permitted reservoirs^31^ is expected to provide durable storage on the order of thousands of years,^32,33^ and so we exclude direct reservoir leakage from this analysis. We describe the methods used to quantify each of these emissions in the following sections.

**Figure 1:**
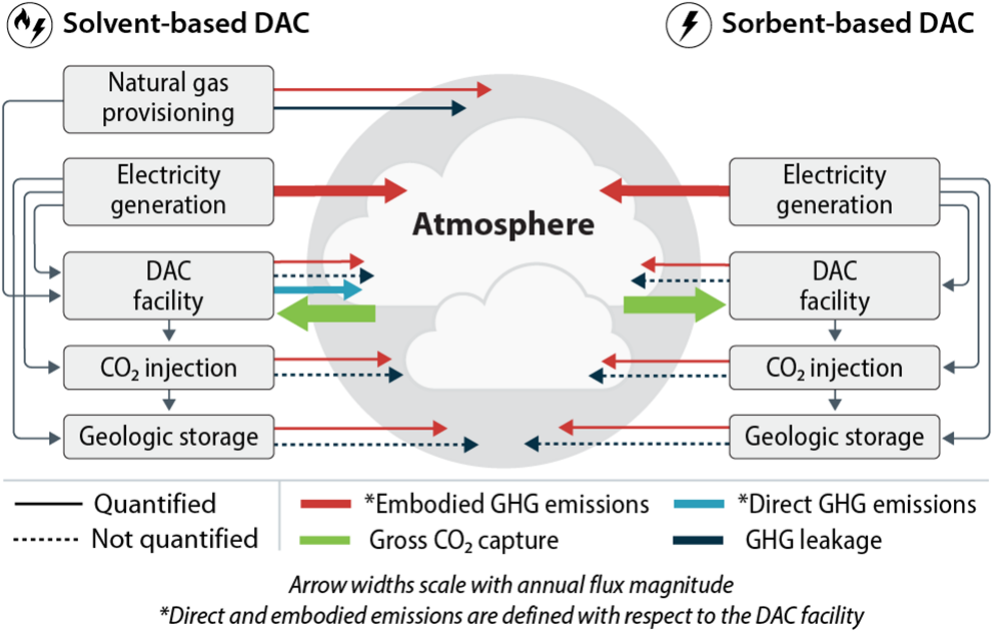
Greenhouse gas fluxes associated with solvent- and sorbent-based DACS. Solid lines represent emissions or removals quantified in this paper, with arrow width representing approximate magnitude. Dashed lines represent fluxes not quantified in this paper, which either are anticipated to be small or lack established quantification methods.

We assess variation in net removal by defining a reference case net removal calculation. The reference case electricity emissions accounting method uses annual volumetric matching with annual average factors derived from net generation-weighted hourly average emissions as modeled in the Mid-Case Standard Scenario^24^ results generated with the ReEDS model.^25^ The reference case applies delivery-area-weighted average factors^34^ for natural gas emissions accounting, and represents a moderate facility operational scenario, described below. The impact of changing the emissions accounting method or facility operational scenario is assessed as a change in calculated net removal relative to this reference case calculation. Note that all assessments of net removal necessarily rely on accounting decisions, such as system boundaries. Although the facility operational scenario is not an emissions accounting decision, it is a major uncertainty with a potentially large impact on net CO_2_e removal.

### DACS Facility Operations and Gross CO_2_ Capture

We use previously developed bottom-up models of sorbent- and solvent-based DACS facilities^27^ with 1 million metric tonne (MMT) CO_2_/year nameplate capacities to calculate hour-by-hour gross CO_2_ capture, electricity use, and natural gas use from hourly, location-specific ambient temperature and relative humidity data^35^ in the operational year 2022. Using 2022 as the basis for this analysis allows for a direct comparison between the most recent publicly available electricity emissions accounting data and modeled electricity emissions accounting data; additional details are given in the next section.

The solvent-based DACS technology modeled here uses both electricity and natural gas,^17^ while the sorbent-based DACS technology relies entirely on electricity, with an electrical heat pump to supply process heat and steam.^36^ The solvent DACS technology includes in situ carbon capture for natural gas combustion emissions.^17^

DACS facilities are capital-intensive, which creates a financial incentive to operate the facilities as close to full capacity as is achievable. However, as with other large chemical processing facilities, DACS facilities require downtime for maintenance and are additionally subject to weather-related efficiency impacts and shutdown requirements.^27^ As a result, a DACS facility’s operational profile varies widely depending on location and local climate. We illustrate this variability and its impact on the calculated net removal by assessing four facility locations, representing a wide range of local climates. These include the warm and dry Central Valley of California, the warm and humid Louisiana coast, the relatively warm and dry western Texas, and the humid and cold eastern part of Wyoming (Table S7 in the SI). Each facility location coincides with a geologic CO_2_ reservoir with available storage capacity, with details in Table S7.^37^ We do not consider economic leakage impacts, which include the possibility that CO_2_ storage now could result in the reservoir reaching capacity, preventing the hypothetical storage of CO_2_ decades in the future.

Solvent-based DACS facilities are expected to encounter difficulties operating at near- or below-freezing temperatures due to a significant increase in solvent viscosity,^38^ which can adversely impact the operational efficiency of the air contactor. The freezing point of the 1 molar KOH solvent used in these facilities is approximately −3.7 °C,^39^ as calculated using the freezing point depression equation. Additionally, air handling operations for both types of DACS technologies may operate less efficiently or cease operating under freezing conditions, especially in high-humidity environments. A DACS facility operator may choose to continue operations at lower efficiencies to maximize CO_2_ gross capture or to shut down during these periods of inclement weather to lower the risk of damaging unit operations. Due to high capital cost as well as operational considerations, such as the need for solvent DACS systems to maintain high operating temperatures for solvent regeneration, the DACS technologies modeled in this analysis are unlikely to be operated in a fully dispatchable manner that could take advantage of time periods when primarily low-carbon generators supply grid electricity, and so we do not model this scenario. Ref 40 demonstrates that a cost-optimizing sorbent or solvent DACS facility powered by variable renewable electricity will expend substantial resources to maximize the DACS onstream factor, including by purchasing expensive energy storage to smooth variability from incoming renewable electricity.

In Table 1, we define three facility operational scenarios based on the expectation that facilities may be shut down during cold temperatures, and the expected need for annual facility maintenance that will occupy 15% of an operational year or approximately 1314 h per year. There are no commercial-scale DACS facilities currently operating from which primary data could be collected. Instead, the 15% annual maintenance time is a conservative value reflecting the assumption that DACS, a relatively new industry, requires roughly twice as much downtime as the oil refinery and petrochemical plant industry plant; annual average plant downtime was 8% in 2024 for one major international oil and gas company.^41^ In the Optimistic operational scenario, the facilities shut down only for this annual maintenance time, which is assumed to occur at the end of the calendar year. In the Reference operational scenario, we assume that temperature-related shutdowns occur when the local temperature drops below 0 °C for more than 15% of the hours in a seven-day rolling time horizon and that the shutdown lasts for 7 days from the beginning of the cold period. We base this assumption on operational challenges faced by one pilot-scale DACS facility in a cold location^42^ and, for solvent DACS, on the need to maintain the solvent above its freezing point. As it is currently unknown whether routine annual maintenance can be performed during temperature-related shutdowns, we include the Pessimistic operating scenario in which facilities shut down during cold temperatures and, separately, for the 15% annual maintenance time, increasing the total shutdown time within a year. Although the solvent freezing point is relevant only for solvent DACS, the impact of cold and humid conditions on air handling operations is the same for both DACS technologies, and so the facility operational scenarios are the same for both technologies. The operational scenarios affect facility onstream factor (the number of operating hours in one year divided by the total hours in one year), gross CO_2_ capture, energy use, water use, and solvent and sorbent material use.

**Table 1:** Definition of the Three Facility Operational Scenarios and the Circumstances under Which Facilities Shut Down Temporarily under Each Scenario^a^

Facility Operational Scenario	Description	Onstream Factor Impact
Optimistic	15% shutdown time for maintenance.	Constant 85% onstream factor across all locations.
	No temperature-related shutdowns.	
	All maintenance occurs at the end of year.	
Reference	Shutdowns for cold temperatures.	Onstream factor varies by location.
	15% shutdown time for maintenance.	
	Maintenance occurs during cold temperature shutdowns.	
	Remaining maintenance occurs at the end of year.	
Pessimistic	Shutdowns for cold temperatures.	Onstream factor varies by location.
	15% shutdown time for maintenance.	
	No maintenance occurs during cold temperature shutdowns.	
	All maintenance occurs at the end of year.	

a Onstream factor is defined as the fraction of hours in the year in which the DACS facility is operating.

Although all simulated facilities have a nameplate capacity of 1 million metric tonnes (MMt) gross CO_2_ capture per year, weather- and maintenance-related shutdowns and efficiency impacts reduce gross annual CO_2_ captured to between 0.8 MMt CO_2_, in the Louisiana solvent case, and 0.3 MMt CO_2_ in the Wyoming sorbent case. Onstream factors and gross CO_2_ capture under the three facility operation scenarios are shown in Table S6 in the SI. For all locations, technologies, and operational scenarios, variability in the hour-by-hour electric load and gross CO_2_ capture is shown in Figures S3 and S4 in the SI.

### Electricity Emissions Accounting

We apply five methods for electricity emissions accounting: average, annual accounting using aggregated, modeled hourly emissions calculated by ReEDS under the Mid-Case Standard Scenario,^24^ which reflects present-day electricity generation technology characteristics and contemporary emissions policies (the reference case), average, annual accounting using primary data^16,28^ (a method commonly used in attributional LCA studies), and average, short-run marginal, and long-run marginal hourly accounting using hourly emission rates calculated by ReEDS^24^ and Cambium.^26^ ReEDS is a capacity expansion model that solves for lowest-cost electricity generation in 134 balancing areas in the contiguous United States; Cambium is a model that postprocesses ReEDS results to calculate short-run and long-run marginal emission factors, among other quantities. For average annual accounting using modeled data, we aggregate hourly emissions generated within each balancing area to the annual level by weighing the emissions produced in each hour by net generation (gross generation minus imports and plus exports) in that hour and dividing the resulting total annual weighted emissions by total annual net generation. These aggregated factors are given in Table S3 in the SI alongside the annual, average factors calculated from primary data. Each balancing area used for this analysis comprises a portion of the state in which it was located, and the modeled emission factors are therefore at a spatial resolution higher than that of the factors based on primary data. Table S7 in the SI gives further details on the location and size of each balancing area. For all accounting methods using modeled emissions, we use electricity emissions data from 2025, the earliest year available from the Cambium model. Raw output from ReEDS and Cambium was obtained from previously executed model runs for the Mid-Case Standard Scenario. No changes to the ReEDS or Cambium input data, parameters, or assumptions were made for this analysis.

For the annual, average accounting method using primary data, we use annual grid mix data, which is available by eGRID region from the U.S. Environmental Protection Agency (EPA).^16^ eGRID grid mix data is published for each calendar year following an approximately two-year lag. At the time of writing, the most recent eGRID data are for 2022, which informed the choice of operational year for this analysis. We combine the eGRID data, which provides the annual mix of generators used in each region (not considering imports or exports, for which the required data are not available^43^), with region- and generator-specific emission factors obtained from the Greenhouse gases, Regulated Emissions, and Energy use in Technologies (GREET) 2023 rev_1 model^28^ to calculate total cradle-to-gate emissions for grid electricity. The region- and generator-specific emission factors are given in the SI, Table S2, and the annual average emission factors calculated from this method are in Table S3. eGRID regions, ReEDS balancing areas, and latitude-longitude coordinates for each location in this study are in Table S7.

We rely on modeled emission factors in the reference case and in all but one of the alternative accounting methods. We do so to ensure a consistent comparison across emissions accounting methods because it is not currently possible to generate corresponding estimates of long-run marginal emission factors using available primary data. While the modeled data may not be a perfect representation of the electric power system in the study locations, the purpose of this study is to illustrate the importance of emissions accounting decisions rather than to produce the most accurate possible estimate of net removal for facilities in the selected locations in 2022.

We model all DACS facilities as fully on-grid, with facility electricity purchased from the local electric grid. Hypothetically, a DACS facility could also operate partially off-grid using either dedicated (colocated, behind-the-meter) renewable generation or a rigorous power purchase agreement (PPA) with a supplier of low-carbon electricity. These alternative powering scenarios replace purchases of bulk electricity from the grid with low-carbon electricity. A rigorous PPA would require an approach similar to the “three pillars” model, which requires (1) new low-carbon supply of electricity, (2) deliverability to the customer facility through the electrical grid, and (3) hourly matching between facility operations and purchased electricity.^44^ Under either alternative powering scenario, emissions associated with purchased grid electricity would be reduced, and the emissions associated with the low-carbon electricity sources would be trivial to model. However, both alternative powering scenarios are likely to be prohibitively expensive due to the nondispatchable nature of DACS technologies and most low-carbon electricity sources. Fully assessing the viability of either alternative powering scenario would require including cost analysis, likely through an emissions-informed dynamic cost optimization model, as in ref 40, which is outside the scope of this analysis.

### Marginal Emission Factors

A DAC facility’s electric load may shift between a short-run load with short-term variability of hours to days and a long-run load with long-term variability of months to years, based on operational decisions and the time since the facility began to operate. (Graphs of each facility’s hourly electric load are given in Figure S3.) Because variation in a DACS facility’s electric load may plausibly be considered short-run, long-run, or both within an operational year, we include both short-run and long-run marginal emissions accounting methods. Short-run marginal emission factors are calculated by assuming that a new electricity load is met entirely by a marginal grid generator dispatched to supply that additional load.^26^ These factors are typically considered most appropriate for transient changes in electricity consumption, such as additional air conditioner use on a hot day. Because the method used to derive short-run marginal emission factors is based on available electricity generators on the grid, short-run marginal emission factors are independent of the magnitude of the new electricity load. Long-run marginal emission factors capture long-term (multiyear) changes in the available grid generators due to new, large, relatively consistent electric loads.^22^ The changes captured by long-run marginal emission factors are structural changes to the grid, as opposed to the operational changes captured by short-run marginal emission factors. However, long-run marginal emission factors carry the implicit assumption that the new load being modeled, in this case, the DACS facility load, is the only new load being added to the grid during the timespan represented. Long-run marginal emission factors imply a counterfactual in which the grid is relatively static, and no new loads are being added, which is unlikely to be true in reality, except over brief time periods. As with the short-run marginal emission factors, the method for deriving long-run marginal emission factors is independent of the new load magnitude. Switching from average to short-run or long-run marginal emission factors may produce substantial changes in calculated net removal from a DACS facility, in a positive or negative direction depending on the facility’s location.

The Cambium model (version 2023), which postprocesses ReEDS results to calculate hourly emissions and costs, provided the short-run and long-run marginal emission factors used in this study, as well as the modeled hourly, average emission factors. These marginal and average emission factors were all calculated under the Mid-Case Standard Scenario, described in ref 24–26 , using these models’ default assumptions surrounding dispatch, investment, and market structure. In all cases, the emissions quantified using Cambium represent cradle-to-gate emission rates, as they include precombustion and combustion emissions as well as transmission, distribution, and efficiency losses.^26^ Due to the different spatial resolutions of the primary data and modeled emission factors, the results are not perfectly intercomparable. In this context, we rely on internally consistent modeled emission factors to evaluate the impact of emissions accounting decisions, including the primary data case for comparison, because it represents common practice at present. Additional discussion on modeling and interpreting marginal emission factors is given in the SI.

### Embodied Emissions for Non-Energy Inputs

We quantify embodied GHG fluxes associated with non-energy inputs (Table 2) using an International Organization for Standardization (ISO)-compliant, attributional life cycle assessment with a cradle-to-gate scope. Reference case embodied flux values are taken from ref 9 and are updated^45,46^ to reflect the same DACS technology models used in this analysis^27^ with the same 1 MMt/year nameplate capacity. To explore the impact on calculated net removals of GHG fluxes embodied in these inputs, we vary the reference values by ±20%.

**Table 2:** Embodied GHG Fluxes for Material and Water Inputs, CO_2_ Transportation and Storage Infrastructure and Operations, and DACS Facility Construction^9,45^ Used in the Reference Case^a^

input	DACS technology	reference case embodied, GHG flux values	units
Water	Solvent	3.43 × 10^–3^	metric tonne CO_2_eq/metric tonne gross CO_2_ captured
CaCO_3_		9.61 × 10^–6^	
KOH		9.85 × 10^–3^	
Amine-based Sorbent	Sorbent	7.19 × 10^–2^	
CO_2_ Transport and Storage	Solvent	6.58 × 10^–2^	metric tonne CO_2_eq/metric tonne CO_2_ stored
	Sorbent	6.58 × 10^–2^	
Facility Construction	Solvent	6.20 × 10^3^	metric tonne CO_2_eq/facility-year
	Sorbent	5.25 × 10^3^	

a An operational lifetime of 25 years is assumed to annualize the facility construction emissions.

See the SI for a description of our approach to estimating life cycle emissions from purchased natural gas, which relies upon delivery-weighted regional average emission factors from ref 34. Note that this study does not incorporate findings of recent comprehensive aerial remote sensing surveys, which find significantly higher methane emission rates in many regions.^47^

## Results and Discussion

### Purchased Grid Electricity Is the Largest Source of Emissions for Most On-Grid DACS Facilities

Figure 2 shows the magnitude of gross CO_2_ captured and GHGs emitted from both DACS technologies across all four locations, calculated using the reference case assumptions and emissions accounting methods described in the previous section. Note that in Figure 2 and throughout this paper positive numbers indicate CO_2_ captured from the atmosphere, and negative numbers represent GHG additions to the atmosphere. Individual GHG emissions are converted into CO_2_ equivalents using 100-year global warming potential factors.^48^ To compute net CO_2_eq removal (black points in Figure 2), we subtract calculated direct and embodied GHG emissions associated with the DACS facility, operations, and infrastructure, as detailed in Figure 1, from the gross CO_2_ captured.

**Figure 2:**
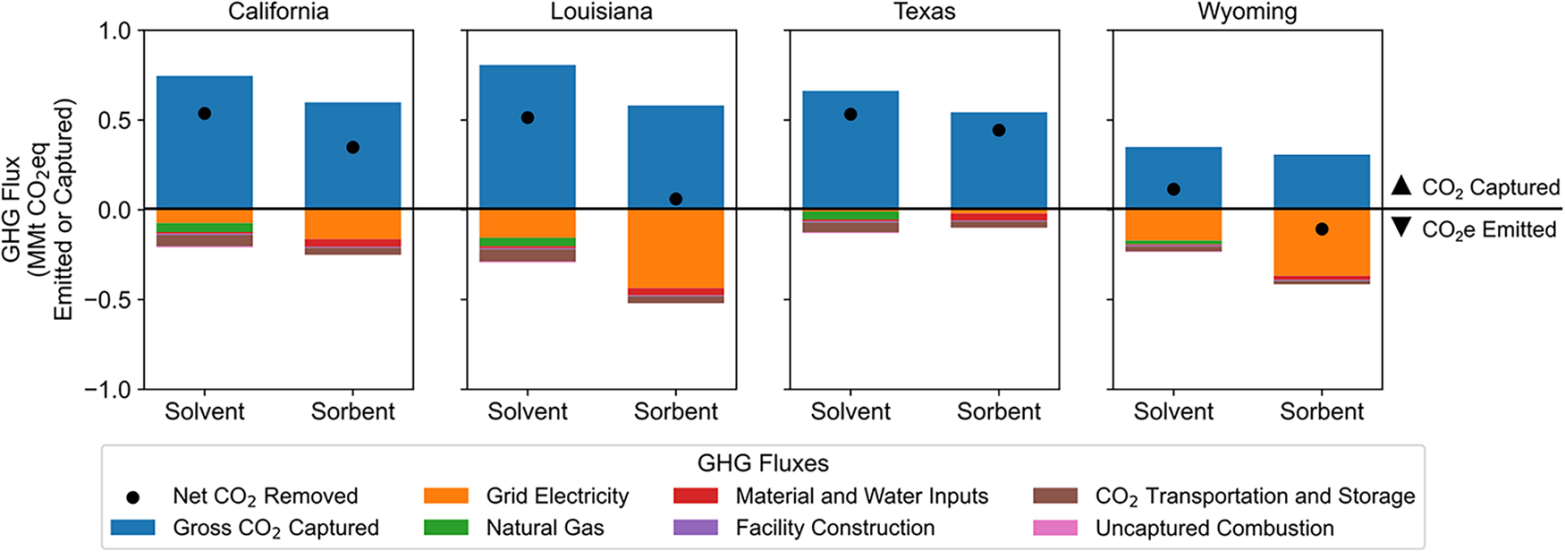
Reference case CO_2_ captured and GHGs emitted for fully on-grid 1 MMt/year nameplate capacity DACS technologies in four U.S. locations, operating in 2022. Positive fluxes are CO_2_ removal from the atmosphere, while negative fluxes are CO_2_eq emissions to the atmosphere. The green “Natural Gas” bar represents only precombustion natural gas-related activities. The solvent DACS facility includes carbon capture for natural gas combustion emissions. “Uncaptured Combustion” fluxes are the remaining CO_2_ emissions to the atmosphere from natural gas combustion.

Figure 2 shows that across three out of four locations evaluated, the largest emission source in the reference case is purchased grid electricity (orange bars), which ranges from 1.5% of gross CO_2_ captured in the Texas solvent facility to 119% in the Wyoming sorbent facility. The interlocation variation in grid electricity emissions is due to the different grid mixes in each location (Figure S2) and different electric loads for each facility (Figure S3 and Tables S8 and S9). Emissions associated with material and water inputs, facility construction, and combined CO_2_ transportation and storage activities range between 11% and 15% of gross CO_2_ captured. Upstream natural gas emissions, which account for emissions intensity from the source regions,^34^ range between 6% and 7% of gross CO_2_ captured for solvent facilities.

### Electricity Emissions Accounting Methods Are Highly Impactful

Annual, average electricity emissions accounting uses the assumption that all electricity purchased throughout the year has a single, constant emissions intensity based on average net generation across the full year for the region in question. This approach is common in current DACS emissions accounting practice.^14,15^ However, the average emissions intensity of grid electricity varies substantially throughout the year and can differ substantially from the short-run or long-run marginal emissions intensity.

Figure 3 highlights this variability in emissions intensity throughout the year alongside the variable electricity demand profiles of sorbent-based DACS facilities in each location. DACS operational electricity demand profiles, in black, include periods of zero electricity demand representing facility downtime for maintenance and weather-related shutdowns. All numbers are aggregated from hourly to daily resolution for readability. Figure S3 in the SI shows electricity demand profiles for all sorbent- and solvent-based DACS scenarios. Total grid electricity emissions are the product of DACS electricity demand curves with electricity emission intensity curves, integrated over the year.

**Figure 3:**
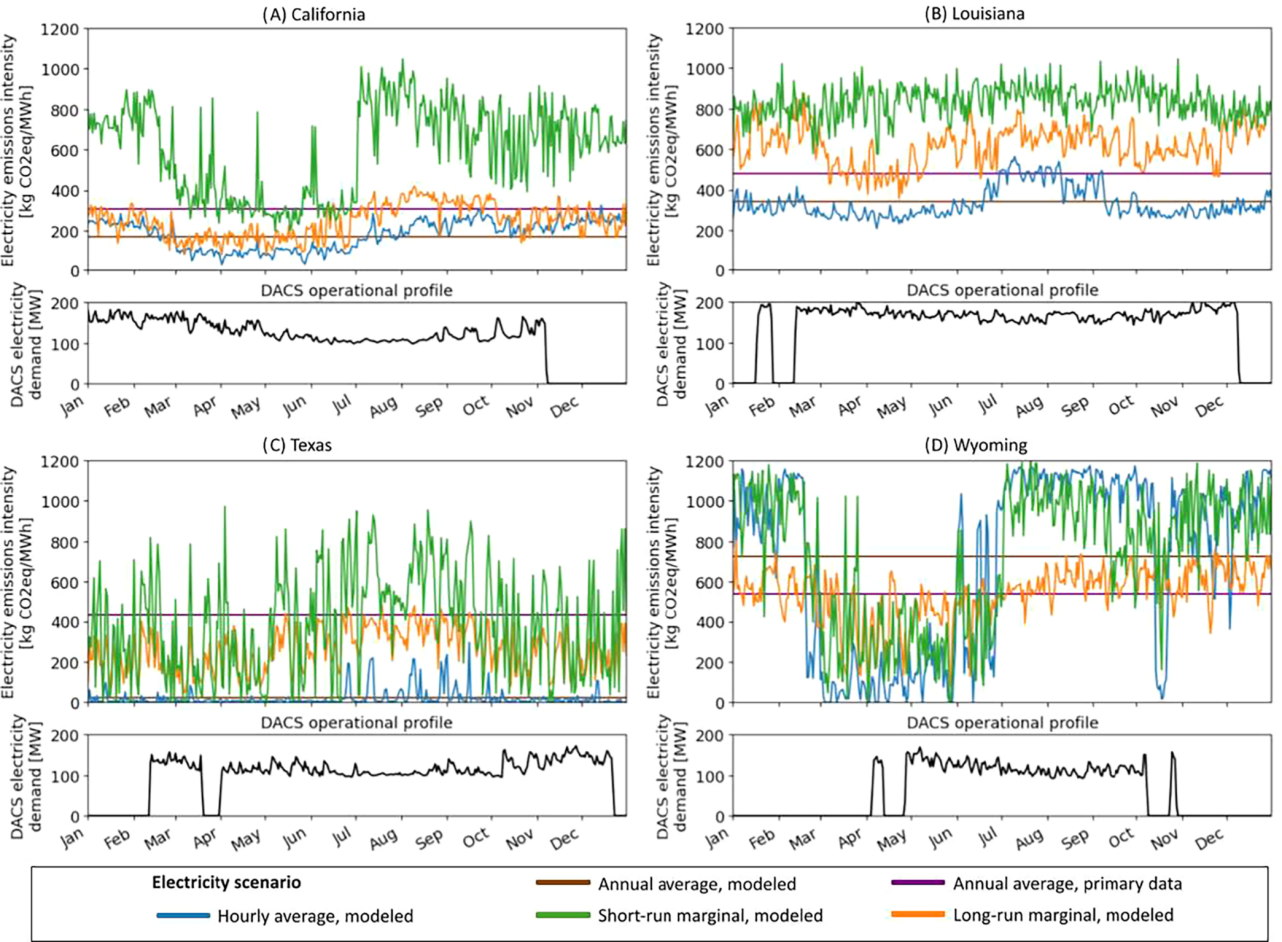
(A–D) Grid electricity emissions intensity across accounting methods and regions. The top subpanels show emissions intensity of electricity using five different methods, aggregated from hourly to daily resolution for readability. The bottom subpanels show reference case daily electricity demand for a sorbent-based DACS facility in each region, incorporating weather impacts on operational efficiency and uptime.

Note that no set of emission factors is consistently higher or lower than all others. For example, in the Texas location, based on a balancing area of 27,677 km^2^, much of the gross within-region generation comes from wind, resulting in an average emission factor of ∼23 kg CO_2_e/MWh. However, dispatchable natural gas generators are typically on the margin in this region, so both short-run and long-run marginal emission factors are higher, on the order of hundreds of kg CO_2_e/MWh. Using the primary data, which quantifies generation at a much coarser spatial resolution, the emission factors for the Texas location represent the highly diversified energy portfolio throughout much of Texas, resulting in a substantially higher estimated average emission factor of 437 kg of CO_2_e/MWh. Primary data at a higher spatial resolution and improved data availability for electricity imports and exports would assist in harmonizing these two numbers. In the Wyoming location, coal typically dominates gross generation, leading to average emission factors that are higher than short-run or long-run marginal emission factors for much of the year. However, wind imports from neighboring regions in late spring and early summer reduce average and short-run marginal emissions during that period.

DACS electricity demand profiles shown in the bottom subpanels of Figure 3 show nontrivial variability in electricity demand over the year, highlighting the potential impact of accounting for time variation in grid emissions intensity. In the reference case, all facilities shut down for cold temperatures and for maintenance, with maintenance occurring during the colder months at the end of the year. This end-of-year period has higher average emissions in the Wyoming location, but lower average emissions in the Texas location. Furthermore, weather-driven DACS efficiency impacts, resulting from temperature and humidity interactions with the chemical processes required to capture CO_2_, result in a 1.9× change in electricity demand throughout the nonmaintenance periods of the year in the California location.

In Figure 4, we assess the impact of electricity emissions accounting methods (indicated by a dashed box), operational scenarios, and uncertainty in embodied emissions for nonelectricity inputs on calculated net CO_2_e removal from fully on-grid solvent-based (Figure 3A) and sorbent-based (Figure 3B) DACS facilities across the four locations. Vertical blue, orange, green, and red lines represent reference net removal for the California, Louisiana, Texas, and Wyoming locations, respectively, and correspond to the black dots in Figure 2. Horizontal bars represent variation from reference case net removal due to an alternate emissions accounting method, facility operational scenario, or uncertainty in embodied emissions for nonelectricity inputs, listed on the *y*-axis of Figure 4. The horizontal bar widths vary only for visibility. As in Figure 2, positive CO_2_e values indicate net removal: more CO_2_ is captured from the atmosphere than CO_2_e is released. The heavy black vertical lines represent zero net removal, at which the amount of CO_2_ removed from the atmosphere is equal to the CO_2_e released to the atmosphere, and are provided for context only.

**Figure 4:**
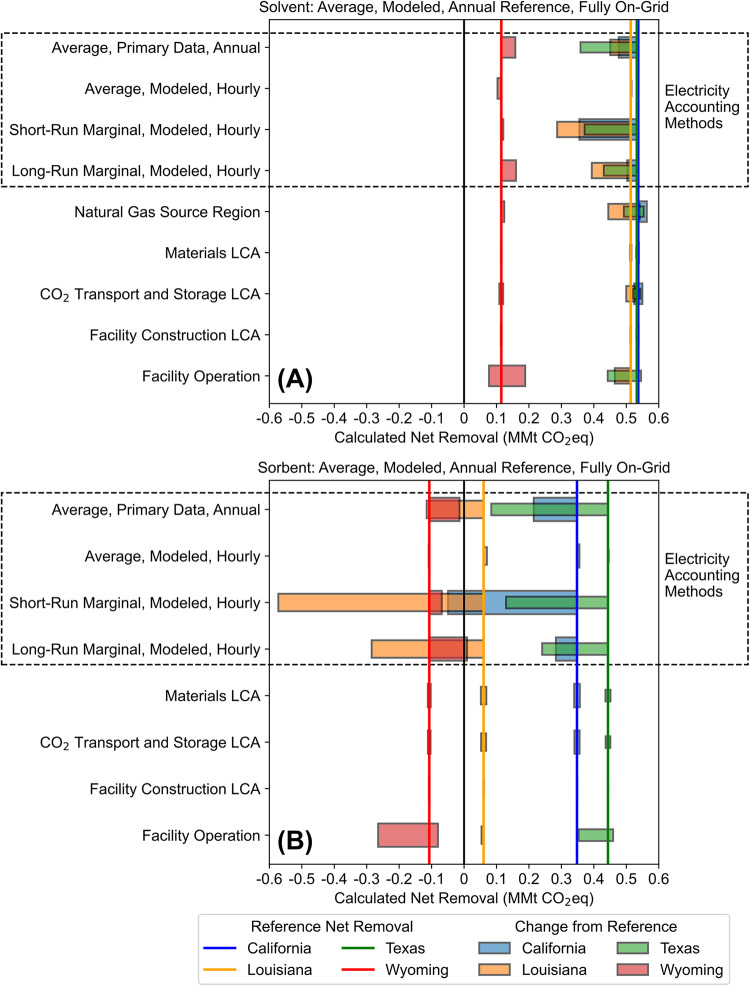
Effect of accounting decisions on calculated net removal. Vertical lines represent reference case net removal for each location. Horizontal bars represent variation from that reference case due to alternate electricity emissions accounting methods, facility operational scenarios, and uncertainty in embodied emissions of nonelectricity inputs. Negative values imply net emissions rather than net removal. The choice of electricity emissions accounting method is more impactful than any other factor evaluated for both solvent (A) and sorbent (B) DACS.

Solvent DACS achieves net removal under all emissions accounting methods and operational scenarios analyzed, with all horizontal bars remaining above the solid vertical black line which indicates the transition from net emissions to net removal. Due to variability in DACS electricity demands driven by local weather conditions and by variation in the local electric grid mixes, sorbent DACS is net-emitting in most Louisiana and Wyoming cases while achieving net removal in all Texas cases and all but one California cases.

There is no single electricity accounting method that results in consistently higher or lower emissions for purchased grid electricity across all cases. Most electricity accounting methods introduced substantial deviation from the reference case, but this variation was not consistent across locations, across technologies, in direction (increasing or decreasing net removal), or in magnitude. Emissions accounting using hourly matching and average modeled hourly emission factors, which capture temporal variations in the emissions intensity of the local grid (Figure 3), can either increase or decrease calculated net removal. The same is true for both short-run and long-run marginal, modeled, hourly emission factors, which capture operational (short-term, hours to days) and structural (long-term, months to years) changes, respectively, in the local grid’s generator mix. Only the calculated net removal for the Wyoming location was relatively unaffected by the electricity emissions accounting method. This is most likely due to the smaller electric load of the Wyoming facilities caused by frequent cold temperatures and subsequent facility shutdowns (Tables S8 and S9).

It is notable that switching from the modeled annual average to hourly average emission factors introduces at most a 17% change in net removal. However, this finding is largely a coincidence for the Wyoming location, as due to persistent cold temperatures, the reference case operational period, roughly April through November, coincides with a representative balance between high- and low-emitting periods. Under different weather conditions, the operational period could shift to include the low-emission March-July period, resulting in emissions substantially lower than the annual average. This highlights that annual average emission factors can produce emissions estimates substantially different from hourly average emission factors.

Using short-run or long-run marginal emission factors causes changes in net removal between −1049% and +40%, and the only location where net removal increased (electricity emissions decreased) when using marginal emission factors was Wyoming. This is due in part to the difference between the average generator mixes in each location, which outside of the Wyoming location contains substantial fractions of nondispatchable, low-emission generators, and the marginal generator mixes, which contain relatively more generation from dispatchable yet higher-emission generators. For the Wyoming location, using marginal instead of average emission factors increases net removal precisely because such a large proportion of the gross generation within the region is coal.

In several cases, the choice of the electricity accounting method determines whether the calculated net removal of CO_2_e for a DACS facility is positive or negative. Within the context of the VCM, this implies that the choice of electricity accounting method could determine whether a DACS facility is able to participate in the VCM through carbon credit sales. For the sorbent facility in Louisiana, using short-run marginal, long-run marginal, or average annual primary data emission factors results in net emissions, causing reductions in net removal of −1049%, −572%, and −291%, respectively. For the Wyoming sorbent facility, using long-run marginal emission factors results in modest net removals of 0.009 MMt CO2e/yr, while all other electricity emissions accounting methods result in a net-emitting facility.

The emissions intensity of purchased natural gas is also a significant driver of calculated net removal for solvent-based DACS (Figure 4). This factor introduces greater variation in estimated net removal in Louisiana and Texas, where facility operators have access to natural gas produced in a wider selection of regions with different ranges of emissions intensity than in California and Wyoming, where there are fewer choices. In the Louisiana location, obtaining natural gas from different sourcing regions that deliver to the facility can change the calculated net removal between −13% and +5%. A list of available natural gas sourcing regions by location^34^ is given in Table S5. The impact of natural gas sourcing region would likely increase with the inclusion of more recent measurement-based methane emission rate^47^ estimates. However, such data are not uniformly available across producing regions within the United States and thus are not incorporated into our analysis.

The uncertainty in embodied emissions associated with facility construction, material use including solvent and sorbent, and water use results in changes in calculated net removal of up to ±14% across technologies and locations. This is smaller in all cases than the changes associated with emissions accounting methods for electricity inputs. This analysis may underestimate the magnitude of embodied emissions from CO_2_ transport and storage: DACS facility locations were chosen to be colocated with geologic storage reservoirs^37^ and the necessary infrastructure build-out was therefore minimal.^9^ For DACS facilities that are not colocated with storage reservoirs, the impact of building out pipelines and other CO_2_ transport infrastructure may be substantial and involve comparatively greater uncertainty. Quantifying embodied emissions from pipeline infrastructure will require allocating total pipeline emissions, including any direct leakage, between all DACS facilities using that pipeline, a nontrivial procedure.

In Figure 4, we also show the impact on the calculated net removal of two alternative facility operational scenarios that change the facility onstream factor, gross CO_2_ capture, and energy requirements. Although facility operation is not an emissions accounting method, differences in how facility-level operational decisions are made have substantial implications for net CO_2_e removal. An optimistic operational scenario, in which all facilities achieve an onstream factor of 85% and shut down only for a single annual maintenance period, increases net removal by as much as 64% in the Wyoming solvent case. A pessimistic scenario in which facilities shut down during periods of cold temperatures and shut down separately for the annual maintenance period reduces net removal for solvent facilities in Louisiana, Texas, and Wyoming by 10% to 33%. However, the impact of facility operations is not consistent across locations: the California site in 2022 did not experience temperatures low enough to trigger any weather-related shutdowns, and all California facilities had an 85% onstream factor across all operational scenarios. The impact of facility operations was strongest in Wyoming, where the frequent cold temperatures result in more shutdown time compared with the other locations.

### Assessment of Accounting Methods for Purchased Grid Electricity

In this analysis, we demonstrate that the choice of emissions accounting method for purchased grid electricity is the single most impactful decision when calculating net CO_2_e removed by a DACS facility. This finding holds across a wide range of climates, electricity grid mixes, facility operational scenarios, and facility powering alternatives. Furthermore, there is no single electricity accounting method that results in consistently higher or lower grid electricity emissions across all cases.

Availability of accurate, up-to-date electricity system data at high spatial and temporal resolutions is a major challenge for emissions accounting for DACS and other energy-intensive facilities with variable electricity demands. The reference electricity accounting method uses annual volumetric matching and average emission factors aggregated from the modeled hourly emissions. Average emission factors are calculated based on the assumption that any electric load, regardless of size, duration, or variability, is supplied by a static combination of all existing grid generators in the region. The implicit assumption is that the electric load being assessed has no effect on the grid or on the emissions associated with purchased electricity. Annual, average accounting for grid electricity aligns with recommendations from several carbon accounting standards,^14,15^ is commonly applied in attributional LCA studies, and as seen in the average, annual, primary data method in this analysis can use high-quality, peer-reviewed, regularly updated data sources.^16,28^ This method, when applied with primary data, is also relatively accessible to nonexperts and is unlikely to be time- or effort-intensive to implement. However, annual average emission factors do not capture subannual variability in either the grid mix or electric load. As seen in Figure 3 and in Figure S3 in the SI, the electric load for DACS facilities can vary substantially within an operational year. The annual accounting method is thus less accurate for DACS than a method with a higher temporal resolution and may disadvantage facilities that shut down for portions of the year or are deliberately operated during times when low-carbon generators are more likely to supply the local grid. An additional source of uncertainty not assessed in this study is the change in emissions intensity of the electric power grid over time. While our study quantifies net CO_2_e removal for facilities operating in 2022, these results would change in different years. Any attempt to evaluate future or lifetime net removal from a DACS facility, which our study does not do, would need to assume a trajectory for the emission intensity of the electric power over the lifetime of the facility, which requires assumptions around future electric grid scenarios.

The spatial resolution of primary data for the annual average method is also relatively coarse, as most eGRID regions cover several U.S. states. Multiple historical electricity emissions data sets exist within the United States, generally at a regional (multiple U.S. states) and annual resolution. Moreover, leveraging historical data necessarily introduces a substantial time lag of one or sometimes multiple years. (Additional discussion of existing historical electricity emissions data sets is given in the SI.) Therefore, although average annual, primary-data-based emissions accounting is currently widely utilized, it may not be the most appropriate method for quantifying emissions associated with DACS and other energy-intensive facilities.

Hourly volumetric matching, in which the electric load of each hour of facility operation is used in accounting, resolves some of the temporal uncertainties of the annual, average method and is being discussed as a future emissions accounting method.^14,49^ To date, there does not appear to be a primary data source for the U.S. that appropriately quantifies hourly grid mixes or emissions for grid electricity. See the SI for additional discussion of the limitations of existing hourly grid data within the U.S.

Marginal emission factors have the advantage of accounting explicitly for the emissions profile of the electricity generated as a consequence of the new demand from the DACS facility. Both short-run and long-run marginal emission factors^22^ are included in this analysis: short-run because of the expected short-term variability in DACS facility electric loads, and long-run because DACS, like any substantial new load on the electric grid, is anticipated to cause structural changes in the grid over months to years. However, the short-run and long-run marginal emission factors as calculated by the ReEDS/Cambium model are independent of load magnitude, and to date, there is no existing method of which we are aware for calculating either set of marginal emission factors as a function of load magnitude.

For a DACS facility with weather-dictated operational constraints and a difficult-to-predict operational profile, neither short- nor long-run marginal emission factors may consistently be appropriate or feasible. Time-weighted marginal emission factors, with weights specified relative to the time since the DACS facility started operation, could allow both operational (short-run) and structural (long-run) changes in the grid to be included. This solution is already being explored in the buildings industry as an alternative to either short-run or long-run marginal emission factors,^50,51^ but would add effort, subjectivity, and uncertainty to the use of marginal accounting methods. See SI for an additional discussion of potential limitations of marginal emission factors. Furthermore, marginal emission factors must rely on modeling assumptions because it is not possible to measure what grid behavior would have been in the absence of an additional electric load, and thus not possible to verify marginal electricity generators or the resulting emission factors against existing primary data. Machine learning tools exist to identify and predict electricity grid emissions intensity in near real time, but they are neither widely deployed nor easily accessible to the public or facility operators.^52^ Because long-run marginal emission factors rely on predictions about the future development of electricity markets, empirical validation is likely not possible, except through retrospective studies conducted years after the fact. The ReEDS and Cambium models, used in this analysis, and similar capacity expansion models,^19^ are intended for evaluating grid-level, long-term trends and may not be fully consistent with present-day generation mixes. Other barriers to using marginal accounting methods include the knowledge, expertise, and time required to generate marginal emission factors. While capacity expansion models are not the only available source of marginal emission factors,^20,21^ the challenge of establishing trusted, standardized calculation methods remains.

Marginal emissions concerns are equally valid for purchased natural gas, as large increases in natural gas demand from solvent-based DACS could spur additional production in locations with a higher or lower emissions intensity. Marginal natural gas system emissions estimation is in its infancy,^53^ and will require substantial additional data collection and methods development before it can be applied rigorously.

Powering a DACS facility partially off-grid using either dedicated (behind-the-meter) electricity generators or a rigorous PPA does not eliminate the challenge of grid electricity emissions accounting. If the dedicated generators are variable, nondispatchable renewables, then given the intermittency of these generators and the currently high cost of long-duration energy storage, powering a DACS facility with mostly dedicated renewable electricity would not currently be feasible or economical. Excess deployment of a combination of dedicated renewable generators to increase capacity utilization would add to capital costs for an already capital-intensive facility, and risk shifting embodied impacts from grid electricity use to other sources, including land use change.^54^ A DACS facility could alternatively reduce use of grid electricity through a rigorous “three pillars” style PPA.^44^ The three pillars are new low-carbon supply of electricity, deliverability to the customer facility through the electrical grid, and hourly matching between facility operations and purchased electricity.^44^ These requirements must be met before the lower-carbon electricity purchased via PPA can be included in emissions accounting. Without all three pillars, there is no guarantee that the electricity contracted for in the PPA is delivered to the facility. This electricity procurement strategy could allow a facility to purchase near-zero emissions of electricity from a suite of generators, enabling more reliable, sustained facility operations than dedicated renewable generation. However, electric utilities in the United States are not yet in a position to offer such PPAs, and doing so may require substantial changes in utility data collection practices, contract structures, and operational capabilities.

The energy emissions accounting challenges highlighted here are not unique to DACS. They apply equally to other emissions-conscious, energy-intensive industries, including low-emission hydrogen, artificial intelligence data center operations, and numerous heavy industrial processes aiming to reduce emissions through electrification. Recent efforts to reduce data center emissions through PPAs and colocation with renewable or nuclear generation have faced significant logistical and regulatory challenges.^55,56^

In conclusion, choosing an emissions accounting method for purchased grid electricity is the single most impactful decision when calculating net CO_2_e removal achieved by a DACS facility. While accounting decisions do not determine physical outcomes, they do determine our best available estimate of emissions caused by electricity consumption at a DACS facility. That a methodological choice, and not directly measurable physical outcomes or intrinsic technology characteristics, is the most impactful component in net CO_2_e removal calculations implies that consensus around the most appropriate emissions accounting method is essential within the VCM to enable DACS participation. High-spatiotemporal-resolution, high-quality, publicly available data sets and models for electricity emissions accounting do not currently exist, and are urgently needed, to enable standardization in emissions accounting methods to more accurately determine the true emissions impacts of DACS and other energy-intensive facilities.

Consensus around an emissions accounting method and the data set or model to support such a method will also be essential for DACS developers to use in siting decisions, economic analyses, and other decision support processes. Without consensus, it is possible to select an emissions accounting method to make a given project artificially appear to be more or less favorable. We identified shortcomings with all of the electricity emissions accounting methods assessed in this work. Development of a more rigorous and consistent method, likely with the aid of regularly updated high spatial and temporal resolution electric power system data, would ease the process of consensus-building and ensure alignment of incentives. However, thoughtful use of existing methods will be necessary in the short term if DACS deployment is to proceed through the VCM or related emissions accounting-dependent mechanisms. Although marginal emission factors may provide additional insight into emissions caused by energy demand from the DACS facility, the reliance on modeling assumptions could challenge the consensus-building process required for their widespread adoption. Whatever method(s) become standard, it will be critical to balance quantitative rigor with usability, ensuring high-quality carbon removal calculations to boost the deployment of effective DACS.

## Supplementary Material



## Notes

Background and input data; additional results; summary of publicly available grid mix data sources; annual average grid mix data for the four eGRID subregions corresponding to DACS facility sites; variation of calculated net removal from a reference case based on optimistic facility operations; variation of calculated net removal from a reference case based on the average, annual electricity emissions accounting method that uses primary data; gross CO_2_ captured and energy inputs for sorbent DAC across four locations and three operational scenarios for the operational year 2022; additional discussion (PDF)
