# Supplementary material for: Energy Emissions Accounting Methods Can Determine Whether Direct Air Capture with Storage Achieves Net Removal
Source: Environ Sci Technol. 2026 Apr 2;60(14):10739–50. doi: 10.1021/acs.est.5c13494 (PMC13085527; doi:10.1021/acs.est.5c13494)
Supplement: Supplementary file 1 [file es5c13494_si_001.pdf]

# Supporting Information: Energy emissions accounting methods can determine whether direct air capture with storage achieves net removal

*Rebecca J. Hanes[1], Keju An[1], Wilson McNeil[2], Yijin Li[1], Isaias Marroquin[1], Soomin Chun[1], Sarah L. Nordahl[2,3], Kimberley K. Mayfield[4], Sarah E. Baker[4], Corinne D. Scown[2,3,5], Evan D. Sherwin[2]*

1 National Laboratory of the Rockies, Golden, CO 80401, USA

2 Lawrence Berkeley National Laboratory, Berkeley, CA 94720, USA

3 Life-cycle, Economics, and Agronomy Division, Joint BioEnergy Institute, Lawrence Berkeley National Laboratory, Emeryville, CA 94608, USA

4 Lawrence Livermore National Laboratory, Livermore, CA, 94550, USA

5 Energy and Biosciences Institute, University of California, Berkeley, Berkeley, CA 94720, USA

Number of pages: 23

Figures: 7

Tables: 9

# Grid mixes and electricity generator emission factors

The grid mix data sources reviewed for use in this analysis are listed in Table S1 with key characteristics. We chose to use the regional- and generator-specific emission factors from Argonne National Laboratory's (ANL) Greenhouse gases, Regulated Emissions, and Energy use in Technologies (GREET) model[1] to calculate the average, annual electricity emissions for reference case net removals. Other data sources and inventory databases were older, less representative, at lower spatial resolutions, or a combination of these factors.

Table S1. Summary of publicly available grid mix data sources.

| | U.S. Average | NERC | eGRID | Balancing Authority | Latest Available Data |
|---|---|---|---|---|---|
| GREET | X | X | | | 2023 |
| U.S. LCI | X | X | X | | 2020 |
| NREL LCA Harmonization | X | | | | 2016 |
| CO2u | X | X | X | X | 2020 |
| U.S. EIA | | | | X | Real-time |
| Electricity Maps | X | | | X | Real-time |

The generator-specific emission factors used in this analysis (Table S2) are at the life cycle scale and represent a cradle-to-gate boundary, where the "gate" is electricity at the consumer (DAC facility, transport system, and injection well). Gate-to-gate GHG emission factors are not appropriate because this small boundary would lead to substantial underestimation of GHG fluxes associated with wind, solar, and hydropower generators, which produce little to no emissions during operations but substantial emissions during manufacturing and deployment. Cradle-to-grave emission factors were not used in this study because generator decommissioning and retirement activities have not been fully studied in the literature, and cradle-to-grave emission factors are not currently available for the full set of generators.

Table S2. Generator-specific emission factors, metric tonne $CO_2$eq/GJ electricity at consumer, sourced from GREET 2023_v1. Calculated from AR6 GWP emission factors and factoring in 5% transmission and distribution losses.

| | *Residual Oil* | *Natural Gas* | *Coal* | *Biomass* | *Nuclear* | *Hydro-electric* | *Wind* | *PV* | *Geothermal* |
|---|---|---|---|---|---|---|---|---|---|
| *U.S.* | 3.06E-01 | 1.49E-01 | 3.08E-01 | 1.60E-02 | 8.29E-04 | 2.07E-03 | 2.83E-03 | 1.04E-02 | 2.79E-02 |
| *ASCC* | 2.85E-01 | 1.77E-01 | 4.29E-01 | 1.60E-02 | 8.29E-04 | 2.07E-03 | 2.83E-03 | 1.04E-02 | 2.79E-02 |
| *FRCC* | 3.14E-01 | 1.42E-01 | 3.11E-01 | 1.89E-02 | 8.29E-04 | 2.07E-03 | 2.83E-03 | 1.04E-02 | 2.79E-02 |
| *HICC* | 3.03E-01 | 2.14E-01 | 3.07E-01 | 1.69E-02 | 8.29E-04 | 2.07E-03 | 2.83E-03 | 1.04E-02 | 2.79E-02 |
| *MRO* | 3.46E-01 | 1.64E-01 | 3.11E-01 | 1.69E-02 | 8.29E-04 | 2.07E-03 | 2.83E-03 | 1.04E-02 | 2.79E-02 |
| *NPCC* | 3.34E-01 | 1.51E-01 | 3.35E-01 | 1.54E-02 | 8.29E-04 | 2.07E-03 | 2.83E-03 | 1.04E-02 | 2.79E-02 |
| *RFC* | 3.14E-01 | 1.50E-01 | 3.07E-01 | 1.62E-02 | 8.29E-04 | 2.07E-03 | 2.83E-03 | 1.04E-02 | 2.79E-02 |
| *SERC* | 3.13E-01 | 1.49E-01 | 3.05E-01 | 1.40E-02 | 8.29E-04 | 2.07E-03 | 2.83E-03 | 1.04E-02 | 2.79E-02 |
| *SPP* | 3.04E-01 | 1.59E-01 | 3.05E-01 | 1.60E-02 | 8.29E-04 | 2.07E-03 | 2.83E-03 | 1.04E-02 | 2.79E-02 |
| *TRE* | 2.99E-01 | 1.51E-01 | 3.08E-01 | 1.30E-02 | 8.29E-04 | 2.07E-03 | 2.83E-03 | 1.04E-02 | 2.79E-02 |
| *WECC* | 2.97E-01 | 1.51E-01 | 3.09E-01 | 1.52E-02 | 8.29E-04 | 2.07E-03 | 2.83E-03 | 1.04E-02 | 2.79E-02 |

The system boundaries for GREET and the National Energy Technology Laboratory's (NETL) CO2u LCA dataset include feedstock production, power plant construction, and power plant operation[1,2]. Both data sources include the complete set of non-renewable and renewable energy carriers listed in Table S2 except for diesel and are maintained and updated annually such that they include the most recent data available. The National Laboratory of the Rockies's (NLR) LCA Harmonization study additionally includes generator end-of-life activities, includes all energy carriers except for diesel, and at the time of writing was last updated in 2021[3]. The U.S. Life Cycle Inventory (U.S. LCI) excludes power plant construction and end-of-life activities and does not include renewable electricity generators, and while the database as a whole is regularly maintained, the electricity data does not reflect current generator technologies[4].

Electricity emissions are also affected by transmission and distribution (T&D) losses, which occur as electricity moves through transmission infrastructure from the power plant to the end user. EIA estimates an annual average T&D loss of 5% for the U.S. which is used to quantify generation relative to demand at the end user. GREET and CO2u emission factors account for the 5% T&D

losses up to the end user, while CO2u, the U.S. LCI, and the NLR LCA Harmonization emission factors account for T&D losses only up to the grid.

Both the U.S. EIA and Electricity Maps derive their data from balancing authority sources, with spatial granularity defined at the balancing authority level. This spatial resolution is limited, as the datasets were not consistent across the U.S. The methodologies employed are insufficiently documented, and access to the Electricity Maps documentation is not consistently free. Furthermore, the generator-specific emission factors used can be outdated and are not fully representative of cradle-to-gate emissions used in this study.

NLR LCA Harmonization emission factors are U.S. averages and thus are not spatially resolved. GREET, CO2u, and the U.S. LCI provide spatially resolved generator-specific emission factors for NERC regions. CO2u and the U.S. LCI emission factors are also available for eGRID regions (a finer spatial resolution compared to NERC regions). CO2u contains a third set of generator-specific emission factors at the balancing authority level. Therefore, CO2u offers cradle-to-gate generator-specific GHG emission factors at the finest spatial resolution of the identified public sources.

## Primary Data and Modeled Grid Mix Comparison

Figure S1 and Figure S2 compare grid mixes for each DAC facility site as obtained from primary data from the Environmental Protection Agency (EPA)[5] and modeled data from ReEDS and Cambium[6,7], respectively. These figures are provided as a point of comparison between the primary-data-based and modeled-data-based annual average emissions accounting methods and underscore the numerical differences between the two methods even when both methods produce average annual emissions factors. Observed discrepancies are partially due to differences in spatial aggregation boundaries, with the EPA data using larger eGrid regions and ReEDS/Cambium using

smaller balancing area regions that have substantially different within-region electricity generation profiles.

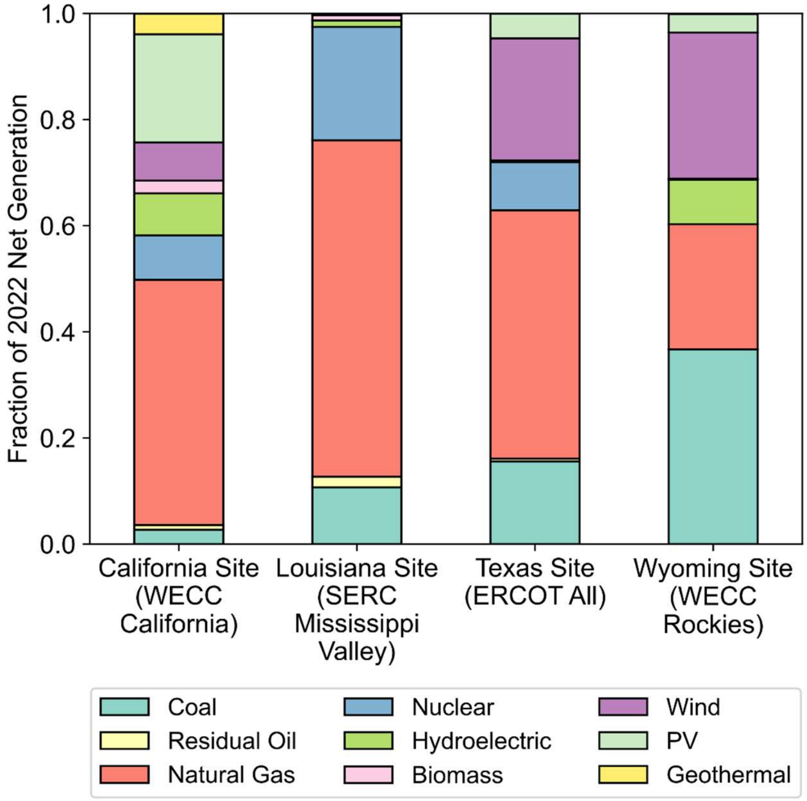


Figure S1. Annual average grid mix data for the four eGRID subregions corresponding to DACS facility sites[5].

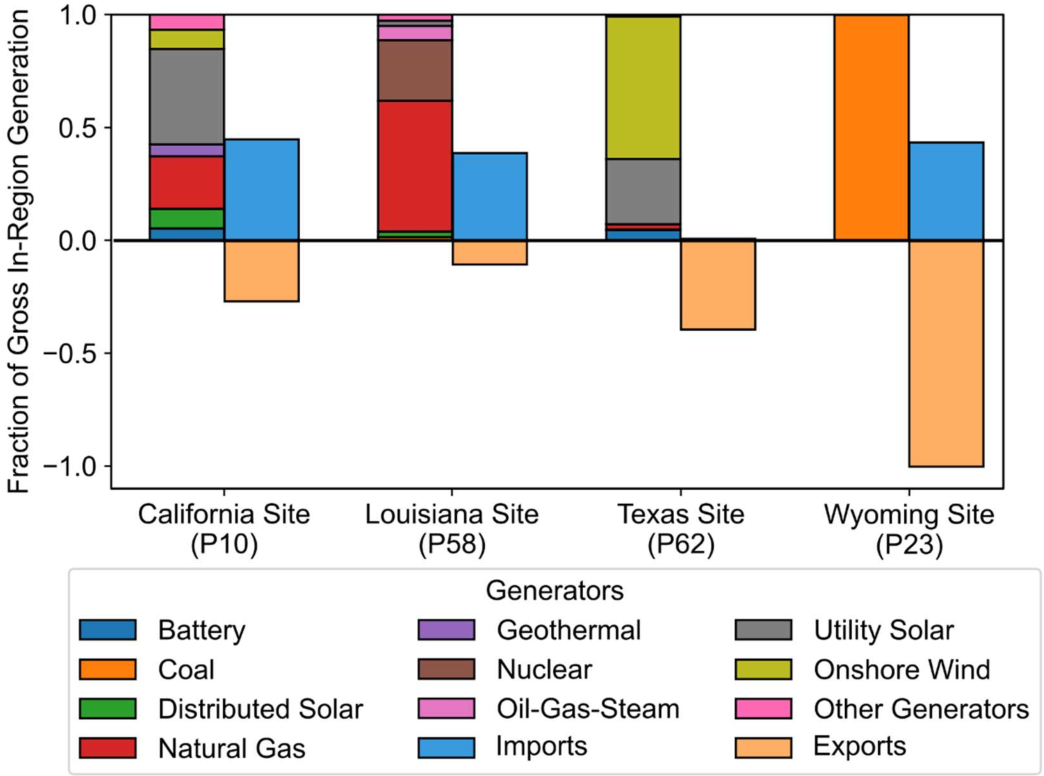


Figure S2. Hourly, average, modeled gross generation, imports, and exports by site, aggregated to the annual level and normalized by total in-region gross generation. This data represents the grid year 2025, the earliest year for which modeled grid emissions data can be generated. The quantities in parentheses denote the ReEDS balancing area[6] in which each facility is located.

Table S3 Annual, average emission factors calculated from hourly, average, modeled emissions (used in the reference case) and from primary data. These factors correspond to the generator mixes shown in the previous two figures.

| | Annual, Average Emission Factors ($kg\ CO_2e/MWh$) | |
|---|---|---|
| Site | Modeled ReEDS and Cambium | Primary Data eGRID and GREET |
| California (P10, WECC California) | 168 | 306 |
| Louisiana (P58, SERC Mississippi Valley) | 342 | 480 |
| Texas (P62, ERCOT All) | 23.0 | 437 |
| Wyoming (P23, WECC Rockies) | 726 | 542 |

Table S4. High-level assessment of feasibility tradeoffs associated with key electricity emissions accounting options. Accuracy of the accounting options are not considered, nor are the economic and technical feasibility of the powering options.

| Method | Transparency | Ease of accounting (assuming required data is available) | Data availability |
|---|---|---|---|
| Annual average, modeled | High | Medium | Model-based |
| Annual average, primary data | High | Easy | Publicly available, but published data are several years old |
| Hourly Average | Low | Easy | Model-based |
| Short-Run Marginal | Depends on model | Medium | Model-based |
| Long-Run Marginal | Depends on model | Medium | Model-based |
| On-site generation | High | Easy | Available |
| Volumetric PPA | High | Easy | Available |
| Three pillars PPA | High | Easy | Model-based |

# Natural Gas Sourcing Regions

Table S5. Summary of natural gas sources and associated emission factors[8].

| Site and Region | Natural Gas Source Regions | Emission Factor Range (mean kg $CO_2$eq/ GJ natural gas delivered) |
|---|---|---|
| California (Pacific) | Green River Conventional, Green River Tight, Permian Shale, Piceance Tight, Uinta Conventional, Uinta Tight | 6.3-11.0 |
| Texas (Southwest) | Anadarko Conventional, Anadarko Shale, Anadarko Tight, Arkoma Conventional, Arkoma Shale, East Texas Conventional, East Texas Shale, East Texas Tight, Fort Worth Shale, Green River Conventional, Green River Tight, Gulf Conventional, Gulf Shale, Gulf Tight, Permian Conventional, Permian Shale, Piceance Tight, San Juan CBM, San Juan Conventional, South Oklahoma Shale, Strawn Shale, Uinta Conventional, Uinta Tight | 6.4-11.0 |
| Wyoming (Rocky Mountain) | Green River Conventional, Green River Tight, Piceance Tight, Uinta Conventional, Uinta Tight | 6.8-24.4 |

| | | |
|---|---|---|
| Louisiana (Southeast) | Anadarko Conventional, Anadarko Shale, Anadarko Tight, Arkoma Conventional, Arkoma Shale, East Texas Conventional, East Texas Shale, East Texas Tight, Fort Worth Shale, Green River Conventional, Green River Tight, Gulf Conventional, Gulf Shale, Gulf Tight, Permian Conventional, Permian Shale, Piceance Tight, San Juan CBM, San Juan Conventional, South Oklahoma Shale, Strawn Shale, Uinta Conventional, Uinta Tight | 4.5-27.7 |

## Additional aspects of the ReEDS model

All ReEDS and Cambium results used in this paper are based on the Mid-Case Standard Scenario generated within the ReEDS model[6,7,9]. This Mid-Case Standard Scenario is characterized by the following assumptions. Cost and performance assumptions for most electricity technologies are based on the 2020 Annual Energy Outlook Reference Scenario. Underlying assumptions are described in detail in reference[6]. Selected important assumptions include:

- Natural gas prices range from $2.50-$4.00 per million British Thermal Units (in $2019 dollars).
- Nuclear power plant lifetimes of 60-80 years without early retirements or lifetime extensions beyond 80 years.
- Multiple state and regional policy mechanisms are included endogenously.

In particular, ReEDS represents California's carbon cap and trade policy as "a cap on electricity-system $CO_2$ emissions from generators either located in California or serving load in the state. Direct $CO_2$ emissions from generators located in California count toward the cap"[6].

## DAC Operational and Site Characteristics

Table S6. Impact of facility operations on onstream factor (number of operational hours divided by total hours in the year) and gross annual $CO_2$ captured.

| Facility | Site | DAC | Onstream | Gross $CO_2$ Captured |
|---|---|---|---|---|

| Operation | | Technology | Factor | (MMt/yr) |
|---|---|---|---|---|
| Optimistic | California | Solvent | 0.850 | 0.746 |
| | | Sorbent | 0.850 | 0.599 |
| | Louisiana | Solvent | 0.850 | 0.804 |
| | | Sorbent | 0.850 | 0.583 |
| | Texas | Solvent | 0.850 | 0.679 |
| | | Sorbent | 0.850 | 0.563 |
| | Wyoming | Solvent | 0.850 | 0.584 |
| | | Sorbent | 0.850 | 0.567 |
| Reference | California | Solvent | 0.850 | 0.746 |
| | | Sorbent | 0.850 | 0.599 |
| | Louisiana | Solvent | 0.850 | 0.807 |
| | | Sorbent | 0.850 | 0.581 |
| | Texas | Solvent | 0.822 | 0.663 |
| | | Sorbent | 0.822 | 0.543 |
| | Wyoming | Solvent | 0.478 | 0.350 |
| | | Sorbent | 0.478 | 0.308 |
| Pessimistic | California | Solvent | 0.850 | 0.746 |
| | | Sorbent | 0.850 | 0.599 |
| | Louisiana | Solvent | 0.762 | 0.730 |
| | | Sorbent | 0.762 | 0.516 |
| | Texas | Solvent | 0.672 | 0.552 |
| | | Sorbent | 0.672 | 0.434 |
| | Wyoming | Solvent | 0.328 | 0.241 |
| | | Sorbent | 0.328 | 0.211 |

### Site characteristics

Table S7. DAC facility site location specifications.

| State | Latitude and Longitude | Reservoir Name and Storage Capacity (MMT $CO_2$)[10] | County | eGRID Sub-Region | ReEDS Balancing Area Name and Size |
|---|---|---|---|---|---|
| California | (35.42, -119.34) | Temblor 1a 38,666 | Kern | Western Electricity Coordinating Council (WECC) California 579,399 $km^2$ | P10 168,313 $km^2$ |
| Louisiana | (30.77, -90.44) | Lower Tuscaloosa 07 6,080 | Tangipahoa | Southeastern Reliability Corporation (SERC) Mississippi Valley 398,941 $km^2$ | P58 103,199 $km^2$ |
| Texas | (31.79, -102.44) | Canyon 3 159,063 | Ector | Electric Reliability Council of Texas (ERCOT) All 715,684 $km^2$ | P62 27,677 $km^2$ |
| Wyoming | (43.85, -105.66) | Madison 1 84,112 | Campbell | WECC Rockies 717,614 $km^2$ | P23 26,033 $km^2$ |

# Additional Results

## Variability in Electricity Load and Gross Capture

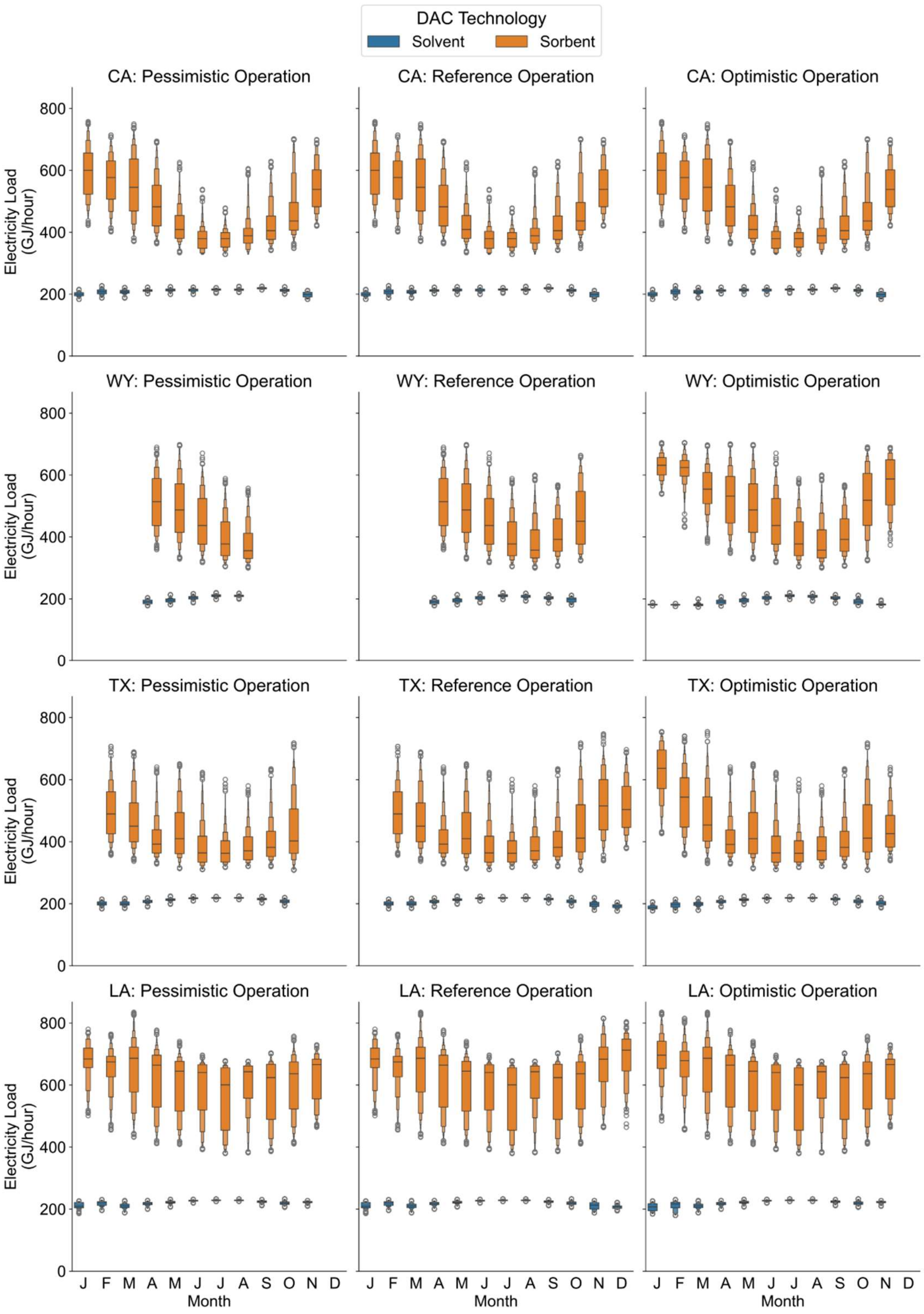


Figure S3. Variability in hourly facility electric load (GJ used per hour) for solvent and sorbent DAC facilities in all four locations and across three operational scenarios, for the year 2022. Periods when the electric load is zero indicate facility shutdowns due to cold temperatures and/or annual maintenance.

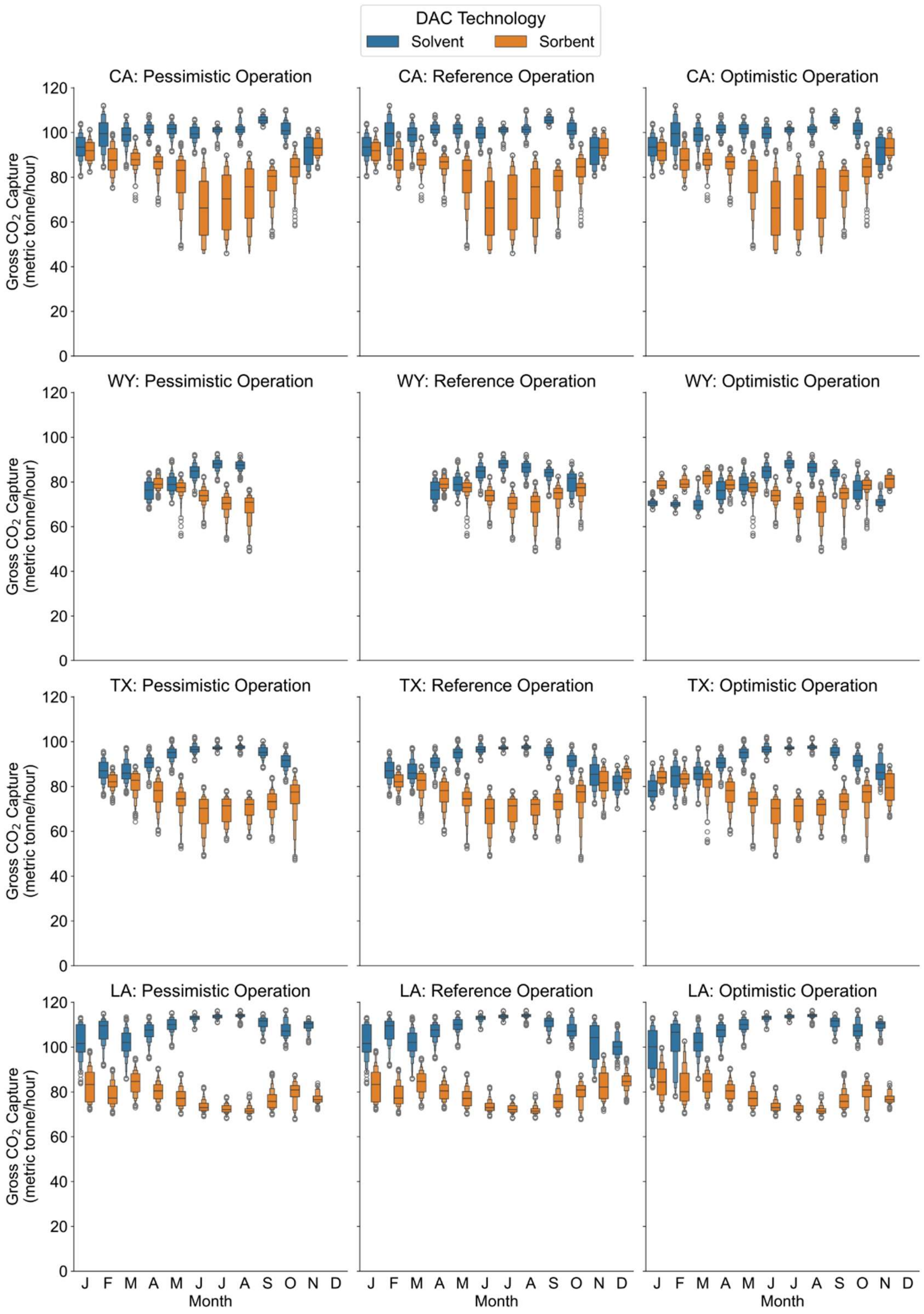


Figure S4. Variability in hourly gross $CO_2$ capture (metric tonnes captured per hour) for solvent and sorbent DAC facilities in all four locations and across three operational scenarios, for the year 2022. Periods when the gross capture is zero indicate facility shutdowns due to cold temperatures and/or annual maintenance.

## Solvent DAC Facility Operations

Table S8. Gross $CO_2$ captured and energy inputs for solvent DAC across four locations and three operational scenarios for the operational year 2022.

| Site | Facility Operations | Gross $CO_2$ Captured (MMt) | Natural Gas (MMGJ) | Electricity (MMGJ) |
|---|---|---|---|---|
| CA | Optimistic | 0.746 | 3.91 | 1.57 |
| | Reference | 0.746 | 3.91 | 1.57 |
| | Pessimistic | 0.746 | 3.91 | 1.57 |
| LA | Optimistic | 0.804 | 4.16 | 1.63 |
| | Reference | 0.807 | 4.18 | 1.64 |
| | Pessimistic | 0.730 | 3.77 | 1.48 |
| TX | Optimistic | 0.679 | 3.56 | 1.55 |
| | Reference | 0.663 | 3.47 | 1.51 |
| | Pessimistic | 0.552 | 2.87 | 1.25 |
| WY | Optimistic | 0.584 | 3.14 | 1.45 |
| | Reference | 0.350 | 1.85 | 0.85 |
| | Pessimistic | 0.241 | 1.27 | 0.58 |

## Sorbent DAC Facility Operations

Table S9. Gross $CO_2$ captured and energy inputs for sorbent DAC across four locations and three operational scenarios for the operational year 2022.

| Site | Facility Operations | Gross $CO_2$ Captured (MMt) | Electricity (MMGJ) |
|---|---|---|---|
| CA | Optimistic | 0.599 | 3.49 |
| | Reference | 0.599 | 3.49 |
| | Pessimistic | 0.599 | 3.49 |
| LA | Optimistic | 0.583 | 4.59 |
| | Reference | 0.581 | 4.58 |
| | Pessimistic | 0.516 | 4.06 |
| TX | Optimistic | 0.563 | 3.33 |
| | Reference | 0.543 | 3.13 |
| | Pessimistic | 0.434 | 2.45 |
| WY | Optimistic | 0.567 | 3.71 |
| | Reference | 0.308 | 1.82 |
| | Pessimistic | 0.211 | 1.28 |

# Additional Results

## Impact of Emissions Accounting Methods under Alternate Reference Cases

In Figure S5, Figure S6, and Figure S7 we re-calculate net removal under alternate reference cases. In Figure S5, the reference case represents the optimistic facility operations scenario, in which all facilities have an 85% onstream factor in all locations. In Figure S6, the reference case represents the pessimistic facility operations scenario, in which facilities shut down for cold temperatures and additionally shut down for regular maintenance. In Figure S7, the reference case uses the average, annual electricity emissions accounting method based on primary data.

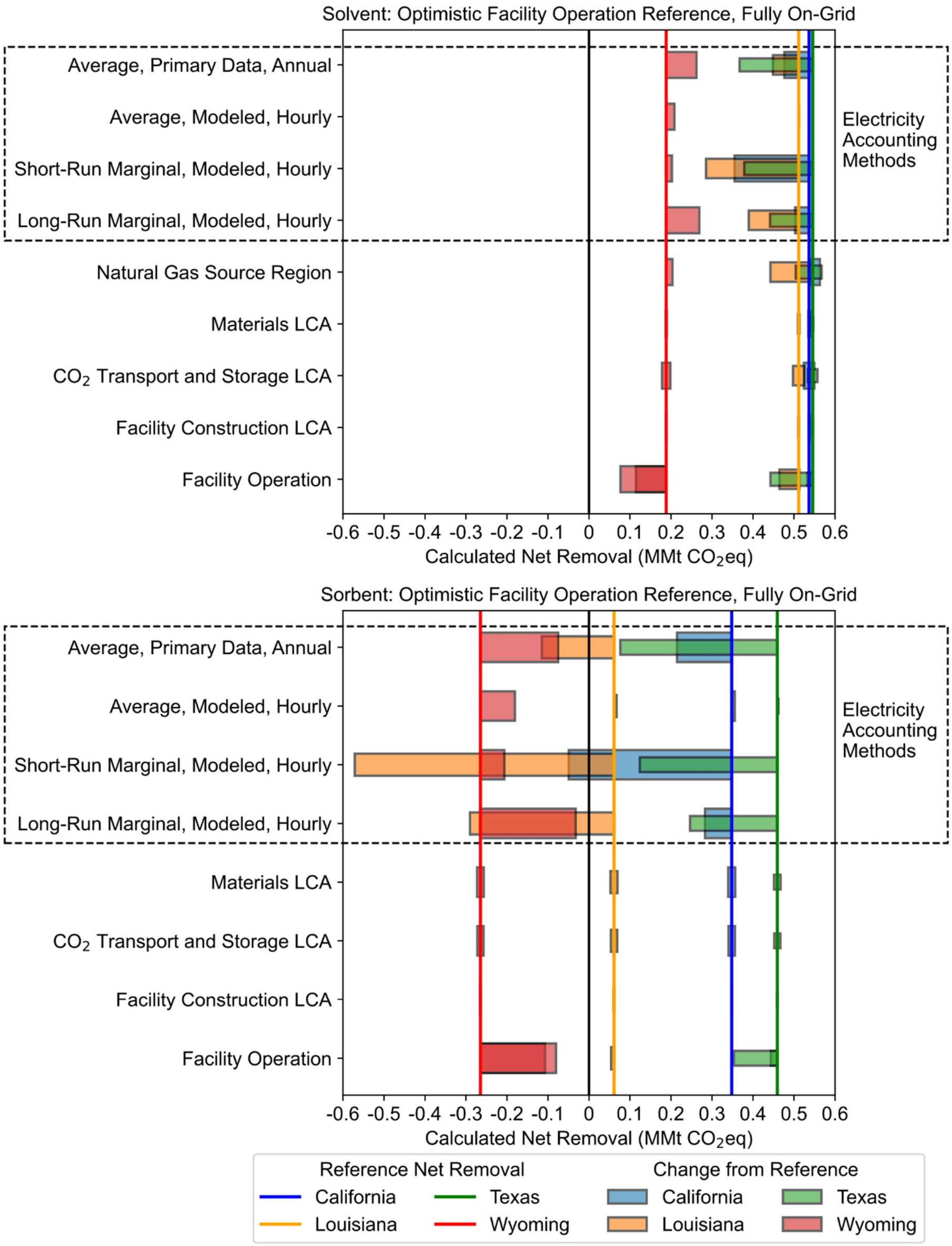


Figure S5. Variation of calculated net removal from a reference case based on optimistic facility operations.

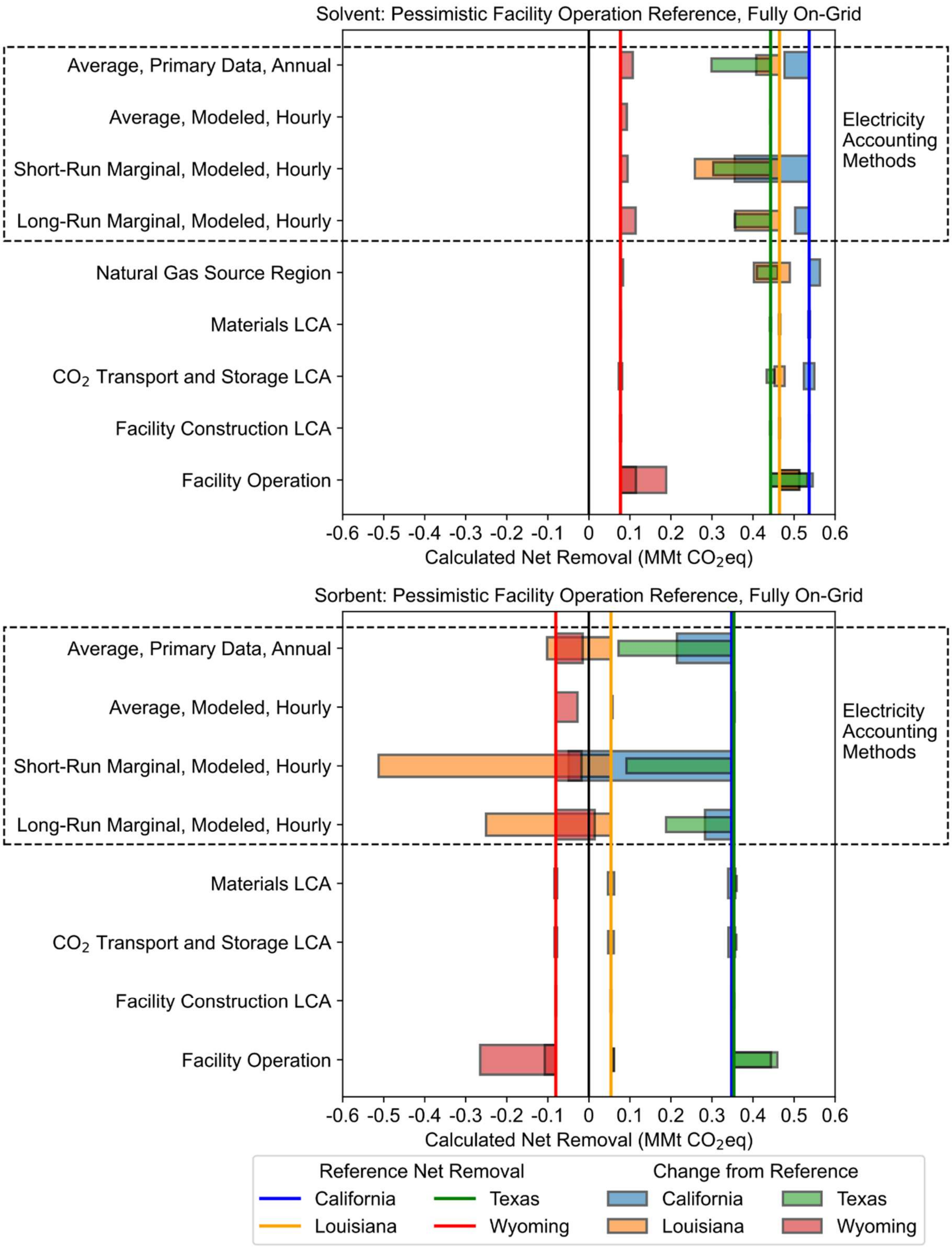


Figure S6. Variation of calculated net removal from a reference case based on pessimistic facility operations.

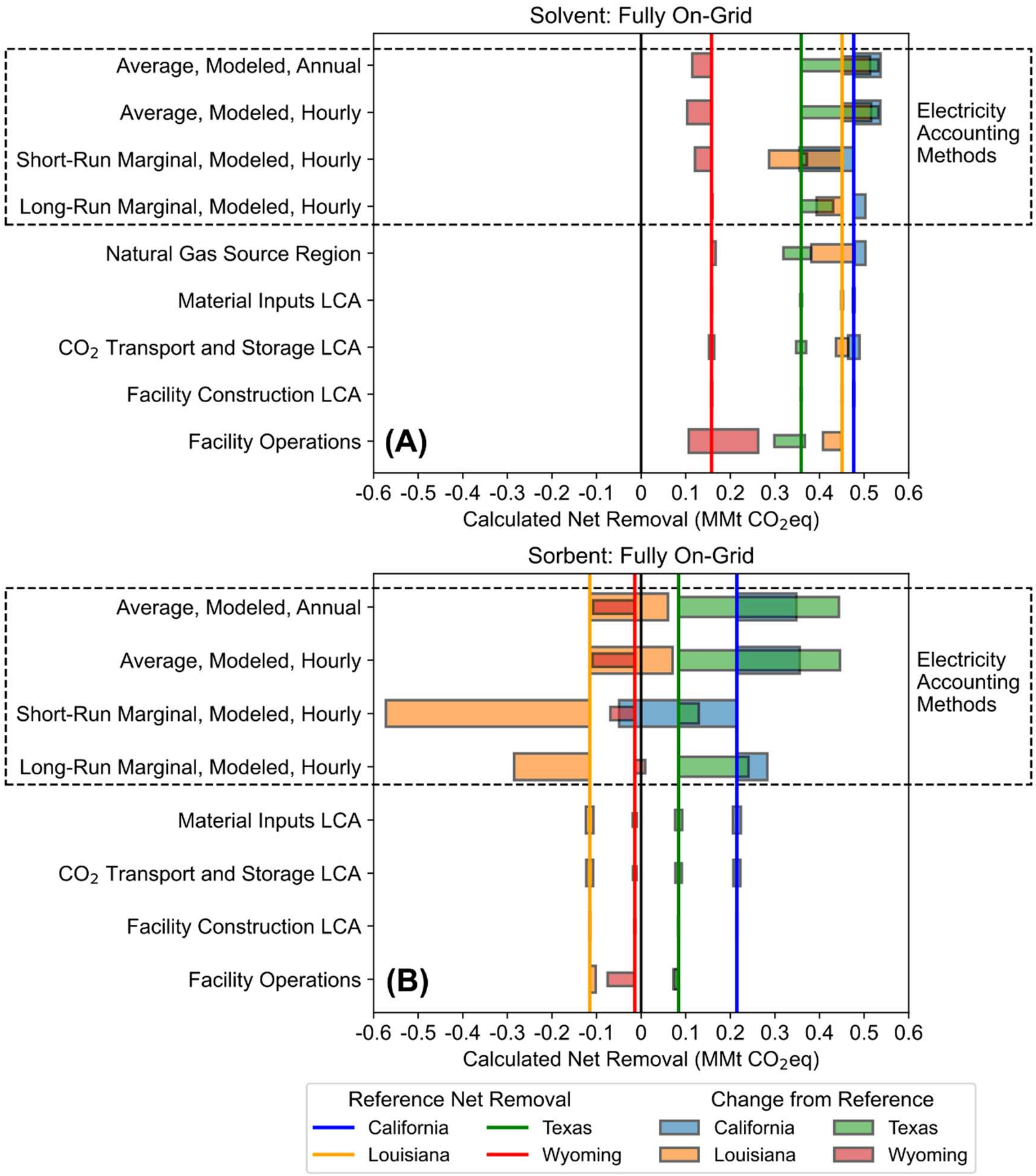


Figure S7. Variation of calculated net removal from a reference case based on the average, annual electricity emissions accounting method that uses primary data.

# Additional Materials and Methods

## Natural Gas Emissions Accounting

Although natural gas-related activities are represented in existing life cycle inventory databases, the available inventories rarely represent the spatial variability of natural gas-related GHG emissions, particularly at the sub-national scale. More importantly, the maturation of new methane

sensing technologies, especially remote sensing via aircraft and satellite, provides a wealth of data that suggest that existing inventories are likely significant underestimates in many cases at all stages of the natural gas value chain, including production, gathering and transmission pipelines and associated compressor stations, as well as gas processing[11–13]. If a facility does not have definite natural gas purchasing information such as a differentiated gas contract, the emissions associated with natural gas provisioning could be either over- or under-estimated. In this analysis, we use a delivery-weighted regional average emission factor for each site[8] in the reference case and assess the variability in calculated net removal due to sourcing natural gas from other regions.

Note that this study does not incorporate findings of recent comprehensive aerial remote sensing surveys, which find significantly higher methane emission rates in many regions[14]. The data source used in this analysis includes cradle-to-consumer estimates of natural gas provisioning emissions for all major oil and gas producing regions in the United States and accounts for non-methane GHGs, including $CO_2$ emissions from gas compression and other combustion processes, which they estimate constitutes between roughly 20% and 45% of total pre-combustion life cycle emissions in most cases (with $N_2O$ accounting for less than 1% of estimated life cycle emissions and $CH_4$ accounting for the remaining majority). Comprehensive leakage surveys have only been conducted in select regions, and to date have not been combined with LCA. Additional data collection would be needed to produce national basin-by-basin measurement-informed emissions inventory with more limited reliance on modeled emissions.

# Additional Discussion

## Low-Carbon Electricity Procurement and Energy Additionality

A key consideration in powering scenarios is clean energy additionality: the principle that the clean energy procured should represent an addition to the renewable energy mix, rather than a

reallocation of existing capacity[15]. The additionality mandate comes from regulatory requirements for "three-pillar" compliance, such as those under the Clean Hydrogen Production Tax Credit (45V)[16], which requires that the renewable electricity used for hydrogen production must be additional, geographically aligned or deliverable, and temporally matched in order to be qualified for appropriate tax incentives. Grid complexity, transmission constraints, and temporal mismatches between generation and consumption can mean that a facility's clean energy purchases do not equate to direct clean energy usage. The "three pillars" are intended to increase the likelihood that a facility procuring low-carbon electricity is receiving that electricity and thereby reducing or eliminating (emissions associated with) their grid electricity consumption.

For low-carbon electricity to be considered additional, it must come from generators that began operating within 36 months prior to the facility starting production. This ensures that the generation capacity used is newly deployed. Deliverability mandates that the additional electricity be generated in the same geological and electric region as the facility purchasing them. This ensures the energy is physically deliverable by reducing or eliminating transmission constraints between the generator and end-use facility. Finally, temporal (hourly) matching requires that the low-carbon electricity be generated during the same hour in which the facility consumes electricity, ensuring that the facility's load is plausibly being met by low-carbon electricity generation. The 45V rule allows annual matching before 2030 for all facilities; after 2030, grid system upgrades that make hourly matching feasible are expected. Power Purchase Agreements (PPAs) and dedicated renewable generation are two alternative low-carbon electricity procurement options available to DACS facilities that are expected to represent additional electricity sources according to current definitions[17]. In contrast, Renewable Energy Certificates (RECs)-style energy procurement does not ensure additionality or "three-pillar" compliance because RECs are

purchased without a direct temporal or spatial link and do not ensure new generator deployment[18]. However, there are challenges, particularly for smaller projects, in obtaining PPA contracts with three-pillar compliance, due to limited financial leverage, the availability of regionally appropriate and timely renewable sources, and complexities in tracking the temporal matching of electricity generation. Additionally, compliance adds transaction costs and administrative burdens, which disproportionately impact smaller facilities and could hinder industry growth.

The additional/non-additional distinction informs the emissions accounting methods that are appropriate for a particular facility's electric load. If the electricity procured is non-additional (such as RECs), then the facility cannot claim to be using low-carbon electricity and therefore emissions accounting should be identical to a fully on-grid facility and based on the regional grid mix. If the electricity is additional, then the facility may use an emissions accounting method that accounts for the lower embodied emissions of the additional electricity. In this case, emissions accounting method should always include life cycle emissions from manufacturing, installing, and maintaining renewable generation systems. For any portion of grid electricity used, which may still include some non-renewable energy sources, the emissions accounting method should again refer to the regional grid mix.

## Average, Short-Run Marginal, and Long-Run Marginal Emission Factors

Average emission factors are defined for a given region and timespan as emissions generated divided by electricity consumption. This method assumes that all electricity demand in the region is met by the same generator mix and assumes the same emission factors for new and variable loads as existing and base loads. An implicit assumption of average factors is that a facility purchasing electricity is equally responsible for emissions from all generators that supplied that electricity.

Short-run marginal emission factors (SRMER) and long-run marginal emission factors (LRMER) are types of consequential emissions accounting methods. SRMER consider that existing loads and new loads will have different effects on the grid. In this model, it is assumed that a singular generator type (e.g., solar, natural gas combined cycle) supplies the additional energy for increases in load in a given region and hour, known as the short-run marginal generator. SRMER typically result in higher carbon dioxide emissions estimates than average factors or LRMER due to the assumption of a single generator type meeting additional demand. This method also has much more variability, from zero emissions if the short-run marginal generator is a renewable source, like solar or wind, or much higher if it is a fossil energy source. Short-run marginal models treat the grid as fixed, whereas LRMER consider that large, persistent changes in demand can optimize the structure and operations of the electricity grid. LRMER assume that a specific mix of generators meet increases in load, in contrast to AER which assume that load is met from all operating generators in the grid mix.

When assessing marginal emissions, a new electricity demand is assumed to be responsible only for emissions resulting from the new electricity generation required to meet the incremental demand on the electricity grid. Two challenges associated with using modeled data are the need to define a prospective counterfactual, e.g. how the grid will change into the future without DAC deployment, and the accessibility of grid models to non-technical experts. As electricity loads evolve due to industry electrification, decarbonization, data center deployment, and policy factors, choosing an appropriate prospective counterfactual is a subjective and non-trivial decision.

The literature has yet to provide a set of specific guidelines for when to use average, short-run, or long-run marginal emission factors (i.e., the size, variability, and duration of the load to warrant marginal over average emissions, or long-run over short-run marginal emissions). Short-run

marginal emissions capture grid operational changes (generators ramping up or down) and are most appropriate for the hours to days following the addition to the grid of a new electric load. For instance, short-run marginal emission factors are applicable the first several days after a DAC facility begins operation or re-starts operation after a shutdown. Long-run marginal emissions represent longer term, structural changes to the grid caused by new generators being deployed or old generators being retired.

## Limitations of existing hourly electricity emissions data

Within the U.S., the only national data source to provide average, hourly grid mix data at any spatial scale is the U.S. Energy Information Administration (EIA), which provides near-real-time operational data reported by some, but not all, balancing authorities[44]. We do not use this dataset here due to quality and spatial representativeness concerns. The U.S. EIA provides the hourly data in an "as is" format, without additional quality checks or reviews, and may therefore contain inaccuracies or omissions. Moreover, the EIA data do not account for electricity imports and exports between balancing authorities, and as such the dataset is not appropriate for emissions accounting.

# References

(1) Argonne National Laboratory. GREET 2023 Rev1, 2023.
(2) Skone, T.; Krynock, M.; Jamieson, M. *NETL CO2U openLCA LCI Database Version 2.1*; None, 1872547; 2022; p None, 1872547. https://doi.org/10.2172/1872547.
(3) National Renewable Energy Laboratory, . *Life Cycle Greenhouse Gas Emissions from Electricity Generation: Update*; NREL/FS-6A50-80580; 2021. https://www.nrel.gov/docs/fy21osti/80580.pdf.
(4) National Renewable Energy Laboratory, . U.S. Life Cycle Inventory, 2024. lcacommons.gov.
(5) U.S. EPA. Emissions & Generation Resource Integrated Database (eGRID), 2024. https://www.epa.gov/egrid.
(6) Ho, J.; Becker, J.; Brown, M.; Brown, P.; Chernyakhovskiy, I.; Cohen, S.; Cole, W.; Corcoran, S.; Eurek, K.; Frazier, W.; Gagnon, P.; Gates, N.; Greer, D.; Jadun, P.; Khanal, S.; Machen, S.; Macmillan, M.; Mai, T.; Mowers, M.; Murphy, C.; Rose, A.; Schleifer, A.; Sergi, B.; Steinberg, D.; Sun, Y.; Zhou, E. *Regional Energy Deployment System (ReEDS)*

*Model Documentation (Version 2020)*; NREL/TP-6A20-78195; National Renewable Energy Laboratory: Golden, CO, USA, 2021. https://doi.org/10.2172/1788425.
(7) Gagnon, P.; Perez, P. A. S.; Obika, K.; Schwarz, M.; Morris, J.; Gu, J.; Eisenman, J. *Cambium 2023 Scenario Descriptions and Documentation*; NREL/TP-6A40-88507; National Renewable Energy Laboratory: Golden, CO, USA, 2024. https://www.nrel.gov/docs/fy24osti/88507.pdf (accessed 2024-10-03).
(8) Littlefield, J.; Rai, S.; Skone, T. J. Life Cycle GHG Perspective on U.S. Natural Gas Delivery Pathways. *Environ. Sci. Technol.* **2022**, *56* (22), 16033–16042. https://doi.org/10.1021/acs.est.2c01205.
(9) Gagnon, P.; Pham, A.; Cole, W.; Awara, S.; Barlas, A.; Brown, M.; Brown, P.; Carag, V.; Cohen, S.; Hamilton, A.; Ho, J.; Inskeep, S.; Karmakar, A.; Lavin, L.; Lopez, A.; Mai, T.; Mowers, J.; Mowers, M.; Murphy, C.; Pinchuk, P.; Schleifer, A.; Sergi, B.; Steinberg, D.; Williams, T. *2023 Standard Scenarios Report: A U.S. Electricity Sector Outlook*; NREL/TP--6A40-87724, 2274777, MainId:88499; 2024; p NREL/TP--6A40-87724, 2274777, MainId:88499. https://doi.org/10.2172/2274777.
(10) National Energy Technology Laboratory. National Carbon Sequestration Database and Geographic Information System (NATCARB), 2024. https://www.netl.doe.gov/coal/carbon-storage/strategic-program-support/natcarb-atlas.
(11) *Meet MethaneAIR | MethaneSAT*. https://www.methanesat.org/project-updates/meet-methaneair (accessed 2024-12-15).
(12) Weller, Z. D.; Hamburg, S. P.; von Fischer, J. C. A National Estimate of Methane Leakage from Pipeline Mains in Natural Gas Local Distribution Systems. *Environ. Sci. Technol.* **2020**, *54* (14), 8958–8967. https://doi.org/10.1021/acs.est.0c00437.
(13) Hmiel, B.; Lyon, D. R.; Warren, J. D.; Yu, J.; Cusworth, D. H.; Duren, R. M.; Hamburg, S. P. Empirical Quantification of Methane Emission Intensity from Oil and Gas Producers in the Permian Basin. *Environ. Res. Lett.* **2023**, *18* (2), 024029. https://doi.org/10.1088/1748-9326/acb27e.
(14) Sherwin, E. D.; Rutherford, J. S.; Zhang, Z.; Chen, Y.; Wetherley, E. B.; Yakovlev, P. V.; Berman, E. S. F.; Jones, B. B.; Cusworth, D. H.; Thorpe, A. K.; Ayasse, A. K.; Duren, R. M.; Brandt, A. R. US Oil and Gas System Emissions from Nearly One Million Aerial Site Measurements. *Nature* **2024**, *627* (8003), 328–334. https://doi.org/10.1038/s41586-024-07117-5.
(15) Gillenwater, M. What Is Additionality? *Discuss. Pap.*
(16) U.S. Department of the Treasury. *U.S. Department of the Treasury Releases Final Rules for Clean Hydrogen Production Tax Credit*. U.S. Department of the Treasury. https://home.treasury.gov/news/press-releases/jy2768 (accessed 2025-01-10).
(17) Haley, B.; Hargreaves, J. *45V Hydrogen Production Tax Credits: Three-Pillars Accounting Impact Analysis*. https://www.evolved.energy/post/45v-three-pillars-impact-analysis (accessed 2024-12-20).
(18) Gillenwater, M.; Lu, X.; Fischlein, M. Additionality of Wind Energy Investments in the U.S. Voluntary Green Power Market. *Renew. Energy* **2014**, *63*, 452–457. https://doi.org/10.1016/j.renene.2013.10.003.
